# ZNF498 promotes hepatocellular carcinogenesis by suppressing p53-mediated apoptosis and ferroptosis via the attenuation of p53 Ser46 phosphorylation

**DOI:** 10.1186/s13046-022-02288-3

**Published:** 2022-02-28

**Authors:** Xiuyuan Zhang, Qijian Zheng, Xiuying Yue, Zhanna Yuan, Jiming Ling, Yanzhi Yuan, Yanying Liang, Aihua Sun, Yuchen Liu, Hui Li, Kaikun Xu, Fuchu He, Jian Wang, Jin Wu, Chunling Zhao, Chunyan Tian

**Affiliations:** 1 School of Life science and Technology, Weifang Medical University, Weifang, 261053 Shandong Province China; 2 State Key Laboratory of Proteomics, Beijing Proteome Research Center, National Center for Protein Sciences (Beijing), Beijing Institute of Lifeomics, Beijing, 102206 China; 3 Tianjin Baodi Hospital, Baodi Clinical College of Tianjin Medical University, Tianjin, 301800 China; 4 School of Public Health, Shandong First Medical University & Shandong Academy of Medical Sciences, Taian, 271016 Shandong China; 5 Shanghai University of Medicine & Health Sciences, Shanghai, 201318 China; 6 Department of Pediatric Surgery, Xinhua Hospital, School of Medicine, Shanghai Jiaotong University, Shanghai, 200092 China; 7 Department of Gastroenterology and Nutrition, Shanghai Institute for Pediatric Research, School of Medicine, Shanghai Jiaotong University, Shanghai, 200092 China

**Keywords:** ZNF498, p53, Phosphorylation, Apoptosis, Ferroptosis, Hepatocellular carcinoma

## Abstract

**Background:**

Dysfunctional p53 signaling is one of the major causes of hepatocellular carcinoma (HCC) tumorigenesis and development, but the mechanisms underlying p53 inactivation in HCC have not been fully clarified. The role of Krüppel-associated box (KRAB)-type zinc-finger protein ZNF498 in tumorigenesis and the underlying mechanisms are poorly understood.

**Methods:**

Clinical HCC samples were used to assess the association of ZNF498 expression with clinicopathological characteristics and patient outcomes. A mouse model in which HCC was induced by diethylnitrosamine (DEN) was used to explore the role of ZNF498 in HCC initiation and progression. ZNF498 overexpression and knockdown HCC cell lines were employed to examine the effects of ZNF498 on cellular proliferation, apoptosis, ferroptosis and tumor growth. Western blotting, immunoprecipitation, qPCR, luciferase assays and flow cytometry were also conducted to determine the underlying mechanisms related to ZNF498 function.

**Results:**

ZNF498 was found to be highly expressed in HCC, and increased ZNF498 expression was positively correlated with advanced pathological grade and poor survival in HCC patients. Furthermore, ZNF498 promoted DEN-induced hepatocarcinogenesis and progression in mice. Mechanistically, ZNF498 directly interacted with p53 and suppressed p53 transcriptional activation by inhibiting p53 Ser46 phosphorylation. ZNF498 competed with p53INP1 for p53 binding and suppressed PKCδ- and p53INP1-mediated p53 Ser46 phosphorylation. In addition, functional assays revealed that ZNF498 promoted liver cancer cell growth in vivo and in vitro in a p53-dependent manner. Moreover, ZNF498 inhibited p53-mediated apoptosis and ferroptosis by attenuating p53 Ser46 phosphorylation.

**Conclusions:**

Our results strongly suggest that ZNF498 suppresses apoptosis and ferroptosis by attenuating p53 Ser46 phosphorylation in hepatocellular carcinogenesis, revealing a novel ZNF498-PKCδ-p53INP1-p53 axis in HCC cells that would enrich the non-mutation p53-inactivating mechanisms in HCC.

**Supplementary Information:**

The online version contains supplementary material available at 10.1186/s13046-022-02288-3.

## Background

Hepatocellular carcinoma (HCC), the most prominent primary liver cancer, is the third leading cause of cancer-related death worldwide [1]. Owing to the limited understanding of its sophisticated molecular pathogenesis, the five-year overall survival rate of HCC is 50–70%. The mechanisms of carcinogen-mediated HCC involve the dysregulation of multiple pathways and factors, including the Wnt/β-catenin pathway, the TP53 signaling pathway, and chromatin regulators [2]. p53 inactivation is one of the most prevalent causes of HCC tumorigenesis and development.

Since its discovery 40 years ago, the p53 protein has been well characterized as a tumor suppressor. In response to different stresses, p53 is activated and regulates a host of cellular processes, including metabolism, apoptotic and nonapoptotic cell death, and migration/invasion, thereby leading to tumor suppression [3]. Mutations in the *TP53* gene are a leading contributor to p53 pathway inactivation in HCC [4]. Recent research has also shown that many HCCs exhibit non-mutation-related p53 inactivating mechanisms [5]. MDM2 is a ubiquitin ligase (E3 ligase) that targets p53, and dysfunction of the MDM2-p53 axis plays a critical role in promoting HCC development and progression [6]. The deregulation of other posttranslational modifications (PTMs) of p53, such as phosphorylation, acetylation and methylation, is also involved in the loss of function of p53 and plays a critical role in HCC tumorigenesis [7, 8].

p53 Ser46 phosphorylation is a principal PTM of p53 [9]. Different cellular stress conditions induce p53 Ser46 phosphorylation, resulting in cell death via apoptosis and ferroptosis. This PTM has been implicated in p53 activation and many kinds of pathophysiology. The substitution of proline 47 with a serine (Ser47) has been shown to decrease p53 Ser46 phosphorylation. This polymorphism impairs the tumor suppressor function of p53, and mice with homozygous or heterozygous expression of p53 (Ser47) are susceptible to the spontaneous development of cancers of diverse histological types, especially HCC [10]. Furthermore, a number of Ser46 kinases, including DYRK2, HIPK2, PKCδ, and p38α, are deregulated in human cancers, which indicates the important role of p53 Ser46 phosphorylation in tumor suppression [9].

Krüppel-associated box (KRAB) domain zinc-finger proteins (KZFPs) constitute the largest mammalian transcription factor/transcriptional regulator family in higher vertebrates [11]. KZFPs are characterized by the presence of an N-terminal Krüppel-associated box (KRAB) domain and an array of C-terminal C2H2 zinc finger (ZF) domains [12]. KZFPs have been shown to play roles in some processes, such as genomic imprinting, cell differentiation, and sexual dimorphism, primarily by repressing transposable elements (TEs) [13–15]. Recently, a growing number of studies have reported the involvement of KZFPs in multiple aspects of tumor biology, and most of these studies demonstrate that KZFPs act as tumor suppressor genes. For example, ZNF516 has been shown to suppress EGFR and inhibit breast cancer growth and metastasis [16]. A tumor suppressor role has also been described for ZBRK1 and ZNF545 [17–19]. Interestingly, only a few studies have focused on the tumor promotion potential of KZFPs [20].

To screen cancer-associated KZFPs, we first compared the mRNA levels of some human KZFPs between tumor and normal tissues using publicly available The Cancer Genome Atlas (TCGA) pancancer datasets and found that ZNF498 mRNA was obviously increased in HCC tissues compared with noncancerous liver tissues. ZNF498, also known as ZSCAN25, is a poorly studied KZFP. Here, we examined the role and underlying mechanisms of ZNF498 in the tumorigenesis and progression of HCC. We demonstrated that the upregulation of ZNF498 expression was associated with advanced tumor grade and a poor prognosis in HCC. ZNF498 promoted diethylnitrosamine (DEN)-induced hepatocarcinogenesis and progression in mice. ZNF498 suppressed p53 transcription by attenuating p53 Ser46 phosphorylation, and it accomplished this by competing with p53INP1 to bind p53. ZNF498 promoted tumorigenesis and progression in vitro and in vivo in a p53-dependent manner. Moreover, immunohistochemistry (IHC) staining analysis of human HCC specimens revealed a correlation between ZNF498 and wild-type p53. ZNF498 suppressed apoptosis and ferroptosis by interfering with p53 Ser46 phosphorylation-mediated transcriptional activity. These findings demonstrate the role of the ZNF498-p53 signaling axis during tumorigenesis and progression and highlight the importance of ZNF498 as a valuable therapeutic target for HCC.

## Methods

### Cell culture and transfection

Hep3B and SMMC7721 HCC cells, L-02 hepatocytes, HepG2 hepatoblastoma cells, HCT115 p53^−/−^ colon cancer cells and HEK293T embryonic kidney cells were maintained in DMEM supplemented with 10% fetal bovine serum (Zhejiang Tianhang Biotechnology, Hangzhou, China). For transient plasmids or siRNA transfection, TurboFect transfection reagent was used following the manufacturer’s protocol (R0532, Thermo Fisher Scientific, Waltham, MA).

### Plasmids, antibodies, siRNA and reagents

Plasmids containing the full-length ZNF498 and its truncated mutants and those containing full-length p53 and its truncated mutants were constructed by PCR. Detailed information is available from the authors upon request. The pLIVE™ vector, which is designed for liver-specific expression and utilizes a chimeric promoter composed of the mouse minimal albumin promoter and the mouse alpha fetoprotein enhancer II (MIR 5320, Mirus Bio Corporation), was selected to construct the liver-specific ZNF498 overexpression vector [21]. The full-length coding sequence (CDS) of *Mus ZNF498* was amplified and cloned into the *Sal*I and *Sac*II sites of the pLIVE vector to generate the pLIVE-ZNF498 vector. For immunoblotting and immunoprecipitation detection of endogenous ZNF498, a rabbit polyclonal antibody was raised by GenScript (Piscataway, USA) against a keyhole-limpet hemocyanin (KLH)-coupled peptide encoding 15 amino acid residues (270–284, GGGSKEKEAKPPQEC) specific to human ZNF498. All other antibodies are listed in Table S1. The siRNAs were purchased from GenePharma (Suzhou, China), and all siRNA sequences are listed in Table S2. The ferroptosis inducer erastin (S7242), RSL3 (S8155), imidazole ketone erastin (IKE, S8877), ferroptosis inhibitor ferrostatin-1 (Fer-1) (S7243), apoptosis inhibitor ZVAD-FMK (S7023), and p53 activator Nutlin-3 (S1061) were purchased from Selleckchem (Houston, USA).

### TCGA dataset

The Zscores of ZNF498 mRNA in HCC were downloaded from the cBioPortal file “data_RNA_Seq_v2_mRNA_median_all_sample_Z scores.txt”. Data on *TP53* mutational status was downloaded from the file “data_mutations_extended.txt” (Table S3).

### Patient tissue specimens

The experiment with patient tissue specimens was authorized by the Human Ethics Committees of Zhongshan Hospital, Fudan University and Cancer Hospital & Institute, Peking University in China. All subjects provided written informed consent.

### Protein extraction

Minced liver tissues were lysed in T-PER buffer (Thermo Fisher) containing protease and phosphatase inhibitors (Thermo Fisher), followed by 1 min of sonication (3 s on and 3 s off, amplitude 25%). The lysate was centrifuged at 16,000×g for 10 min, and the supernatant was collected as a whole-tissue extract. The protein concentration was determined using the Bradford assay.

### Tissue microarrays and IHC

To verify the ZNF498 and p53 expression levels, tissue microarrays (TMAs) containing 86 pairs of HCC samples and their corresponding nontumorous tissues, in addition to 6 HCC tissues, were evaluated by Shanghai Outdo Biotech Co., Ltd. (Shanghai, China). IHC was performed using the avidin-biotin complex method (Vector Laboratories, Burlingame, CA) and included heat-induced antigen retrieval procedures. The samples were incubated with polyclonal antibodies against ZNF498 (1:1500 dilution) and p53 (1:1000 dilution) at 4 °C for 18 h. Quality assessment was performed on each batch of slides by including a negative control in which the primary antibody was replaced with 10% normal goat serum to preclude nonspecific binding. The staining was assessed by pathologists who were blinded to the sample origins and patient outcomes. The widely accepted German semiquantitative scoring system was used to score the staining intensity and extent of staining in different areas. Each specimen was assigned a score according to the intensity of the nucleic staining (0 for absent, 1 for weak, 2 for moderate and 3 for strong) and the extent of the staining (0–5% = 0, 6–25% = 1, 26–50% = 2, 51–75% = 3 and 76–100% = 4). The final immunoreactivity score was determined by multiplying the intensity score by the extent of the staining score, with the final score ranging from 0 (minimum score) to 12 (maximum score). A ZNF498 expression score < 8.0 was defined as low expression, and ≥ 8.0 was considered high expression.

### Virus production and generation of stable cell lines

The ZNF498 lentiviral expression vector was constructed by inserting the CDS of ZNF498 into the pLV-Neo vector (VL3002, Yingmaoshengye Biotechnology, Beijing, China). The ZNF498-knockdown lentiviral expression vector was constructed by inserting a shRNA sequence targeting human ZNF498 into the pLVshRNA-EGFP (2A) Puro vector (VL3103, Yingmaoshengye Biotechnology). The p53-knockout lentiviral expression vector was constructed by inserting a sgRNA sequence that targeted human p53 into the LentiCRISPRv2 vector (#52961, Addgene). Stocks containing lentiviral particles were generated as previously described. The cells were inoculated in culture dishes containing the same volume of retroviral supernatant and then incubated for 24 h. Then, the medium was exchanged with fresh medium. The cells were screened with G418 or puromycin after infection for 3 days, and after 10 days, single clones were selected and assessed by Western blotting. The shRNA sequences were as follows: ZNF498: 5′-AGCGCACCATCACATCTAATT-3′, and nontargeting control, 5′-TTCTCCGAACGTGTCACGTTT-3′. The sgRNA sequence for p53 was 5′-CGTCGAGCCCCCTCTGAGTC-3′.

### Cell counting Kit-8 (CCK-8) cell proliferation assay

Cells were seeded in a 96-well plate in 0.1 mL of medium. Cell proliferation was measured at 450 nM using 10 μL CCK-8 reagent (CK04, Dojindo CO. Ltd., Kumamoto, Japan) every 24 h. The data represent the average of three independent experiments.

### Colony formation assay

For the colony formation assay, after transfection, 2 × 10^3^ viable cells were plated in six-well plates in triplicate and maintained in complete medium for 15 days. The cells were fixed with 4% polyoxymethylene and stained with 0.1% crystal violet.

### In vivo xenograft mouse model

The experimental procedures in nude mice were approved by the Animal Care and Use Committee of the Academy of Military and Medical Sciences. BALB/c nude mice (6 weeks old, 18.0 ± 2.0 g) were obtained from Vital River Laboratory (Beijing, China). Cells with stable knockdown (4 × 10^5^ per mouse) or overexpression (2 × 10^5^ per mouse) of ZNF498 were subcutaneously injected into the right flanks of the mice, the corresponding control cells were injected into the left flanks. Tumor size was measured every 3 days and converted to volume according to the formula V (mm^3^) = (a × b^2^)/2, where a and b are the largest and smallest diameters, respectively. All animals were killed 30 days after inoculation, and the transplanted tumors were removed and fixed for further study.

### Generation of a liver-specific ZNF498-overexpressing mouse model

The experimental procedures in mice were approved by the Animal Care and Use Committee of the Academy of Military and Medical Sciences. Male C57BL/6 mice were purchased from Vital River Laboratory (Beijing, China). The pLIVE (control) and pLIVE-ZNF498 vectors (5 μg/mouse) were delivered to the mouse liver using the hydrodynamic tail vein injection procedure according to the instructions provided by the manufacturer. To detect ZNF498 expression in the liver, total RNA was extracted from mouse livers using TRIzol reagent 48 h after high-pressure tail vein injection, and quantitative PCR was performed.

### DEN-induced HCC and quantification of tumor burden

The experimental procedures in mice were approved by the Animal Care and Use Committee of the Academy of Military and Medical Sciences. Male C57BL/6 mice were purchased from Vital River Laboratory (Beijing, China). Mice that died during the experiments were excluded from the final analysis. Briefly, 18-day-old male C57BL/6 J mice were intraperitoneally injected with a single dose of the chemical carcinogen DEN (25 μg/g body weight). When the mice weighed 18–22 g (approximately 40 days old), the pLIVE (control) and pLIVE-ZNF498 vector DNA constructs (5 μg/mouse) were delivered to the liver using hydrodynamic tail-vein injection. After injection, the mice were housed under specific-pathogen-free (SPF) conditions and monitored regularly. All mice were sacrificed at 24 or 44 weeks of age. Tumors > 1 mm in diameter were counted and measured. Conventional hematoxylin and eosin (H&E) staining was performed to evaluate signs of malignancies.

### Coimmunoprecipitation (co-IP) and Western blotting

Co-IP and Western blotting were performed as previously described [22].

### In vitro glutathione S-transferase (GST) pulldown assay

Bacteria-expressed GST or GST-ZNF498 proteins were immobilized on glutathione-Sepharose 4B beads (17–0756-01, GE Healthcare) for 4 h and washed with 1 mL of PBS. Then, the beads were incubated with His-p53 expressed in *Escherichia coli* BL21 and purified with Ni-nitrilotriacetate-agarose beads (013771/34220, CWBIO) for 4 h at 4 °C. The beads were washed with GST elution buffer (50 mM Tris-HCl, 10 mM reduced glutathione, pH = 8.0), and the proteins were eluted and then subjected to Western blotting.

### Gene reporter assays

For the reporter assay, 2 × 10^4^ cells were seeded on 24-well culture plates. After 18 h, the firefly reporter construct pG13-Luc (pG13L, containing 13 tandem repeats of p53-binding sites), a gift from Bert Vogelstein (Johns Hopkins Oncology Center), was transfected into HepG2 cells with other expression plasmids; the pRL-TK Renilla luciferase reporter plasmid (E2241, Promega, Madison, USA) served as an internal control to normalize for transfection efficiency. After 48 h, the cells were harvested, and the firefly and Renilla luciferase activities in the lysates were determined using a GloMAX 96-microplate luminometer (Promega) and a dual-luciferase reporter assay system following the manufacturer’s protocol (E1980, Promega). Each transfection was performed in triplicate, and the experiments were repeated three times.

### RT–PCR and quantitative PCR

RT–PCR and quantitative PCR were performed as previously described [23]. Detailed information on the primers for each gene is listed in Table S4.

### Apoptosis analysis

To analyze the apoptosis rate, 2 × 10^5^ cells were cultured in 6-well plates in triplicate. At 36 h after transfection, cells were stained with Annexin V/propidium iodide (PI) staining kit (Lianke, AP101–100 kit) and analyzed with a flow cytometer.

### TUNEL, lipid peroxidation, glutathione (GSH) and reactive oxygen species (ROS) assays

TUNEL-positive cells were stained using a TUNEL apoptosis assay kit, the concentration of malondialdehyde (MDA) was assessed using a lipid peroxidation assay kit, and the intracellular GSH and ROS levels were assessed using a GSH colorimetric assay kit. All of these reagents were purchased from Solarbio (Beijing, China) and used according to the manufacturer’s instructions.

### Statistical analysis

Statistical analysis was performed with SPSS 18 and GraphPad Prism 5.0. The data are presented as the mean ± SD of three independent experiments. ANOVA with Dunnett’s multiple comparisons test was used for studies with comparisons among multiple (> 2) groups, and Student’s t test was used for studies with comparisons between two groups. A chi-square test was used to analyze the correlations between ZNF498 and the clinicopathological parameters. Survival was analyzed using the Kaplan–Meier method, and the difference between the survival curves was analyzed using the log-rank test. Differences with *P* > 0.05 were considered no significance (*ns*), and those with *P* < 0.05 were considered statistically significant. * indicates *P* < 0.05, ** indicates *P* < 0.01, and *** indicates *P* < 0.001 in all figures.

## Results

### ZNF498 is highly expressed in human HCC tissues

**Fig. 1:**
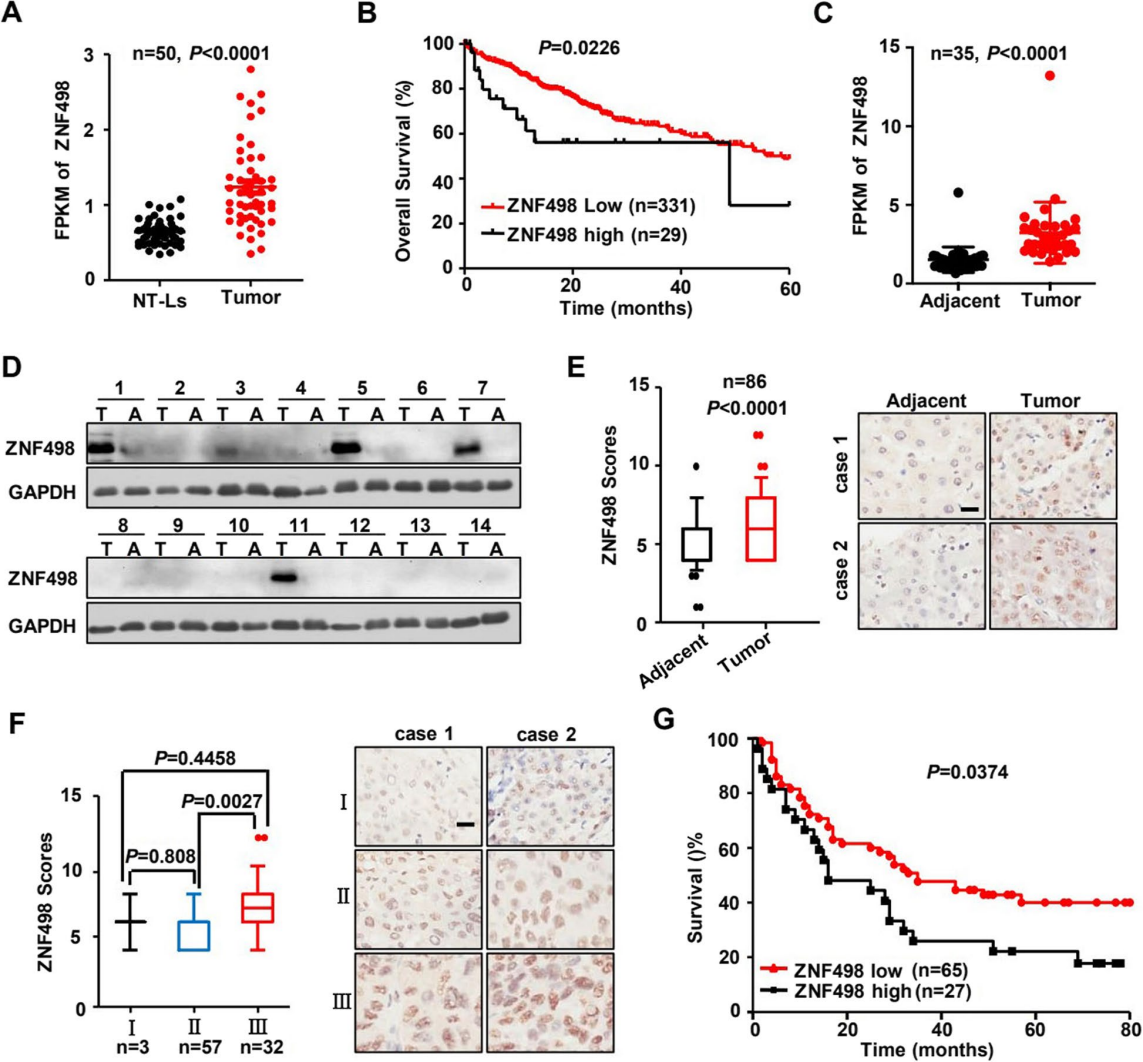
ZNF498 is highly expressed in human HCC tissues. **A** ZNF498 mRNA levels in 50 paired human HCC specimens and adjacent normal tissues from the TCGA database were analyzed. **B** Kaplan–Meier plots of the overall survival of patients with low and high expression levels of ZNF498 mRNA in tumor tissues (cut off =1.1) from the TCGA datasets. **C** ZNF498 mRNA levels in 35 paired human HCC tissues and adjacent normal tissues were analyzed using RNA-seq data available online. **D** ZNF498 protein levels in 14 pairs of HCC and adjacent normal tissue samples were detected by Western blotting. T, tumors; A, adjacent normal tissues. **E** Statistical analysis and representative images of ZNF498 expression in 86 paired HCC tissues and adjacent normal tissues as detected by IHC. **F** Statistical analysis and representative images of ZNF498 expression in HCC tissues (*n* = 92) with different histological grades as detected by IHC. **G** Kaplan–Meier survival curves according to ZNF498 expression level in HCC tissues (n = 92) as detected by IHC. Scale bars, 20 μm. *P < 0.05; **P < 0.01; ***P < 0.001; ****P < 0.0001

To screen cancer-associated KZFPs, we first compared the mRNA levels of some human KZFPs between tumor and normal tissues using TCGA datasets and found that ZNF498 mRNA expression was obviously increased in HCC tissues compared with noncancerous liver tissues (Fig. 1A). In the TCGA HCC cohort, overall survival was significantly lower in patients with high ZNF498 mRNA levels than in those with low ZNF498 levels (Fig. 1B, Table S3), indicating that ZNF498 is a potential HCC-associated KZFP. Additionally, the expression analysis with online available RNA-seq data (35 paired tumor and adjacent normal tissues of clinical early-stage HCC) showed consistent upregulation of ZNF498 mRNA levels in HCC [24] (Fig. 1C). To examine the ZNF498 protein level, we used a specific antibody against ZNF498 (Supplementary Fig. S1) and subsequently performed Western blotting in 14 pairs of HCC and adjacent normal tissue samples. The results confirmed the protein overexpression of ZNF498 in HCC tissues compared with adjacent normal tissues (Fig. 1D). We further evaluated the protein expression of ZNF498 by IHC of 86 matched pairs of HCC and adjacent normal tissues and 6 additional HCC tissues. The level of ZNF498 was increased in liver cancer tissues compared with normal liver tissues (Fig. 1E) and was lower in low histological grade tumors than in high histological grade tumors (Fig. 1F). Furthermore, the correlations between ZNF498 overexpression and clinical parameters in HCC patients were assessed, and the statistical analysis showed that ZNF498 protein expression was also significantly correlated with histological grade (*P* = 0.003, Table S5). Importantly, a follow-up analysis of patient survival demonstrated that patients with tumors expressing higher ZNF498 levels had significantly poorer survival than those expressing low ZNF498 levels (Fig. 1G).

### ZNF498 promotes DEN-induced hepatocarcinogenesis and progression in mice

**Fig. 2:**
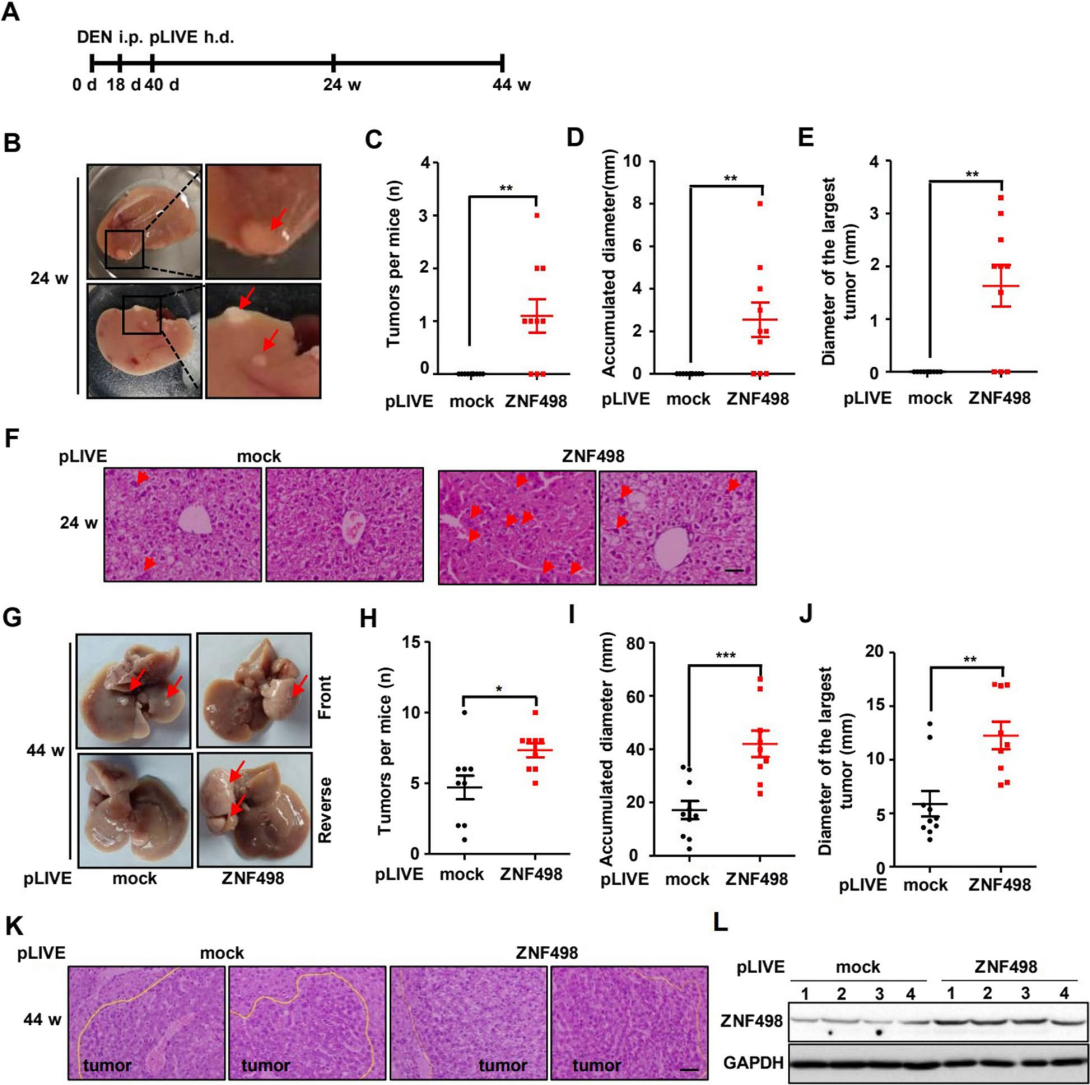
ZNF498 promotes DEN-induced hepatocarcinogenesis and progression in mice. **A** Male C57BL/6 mice were treated with DEN at 18 days old, and the constructs of pLIVE (control) and pLIVE-ZNF498 vector DNA (5 μg/mouse) were delivered to the mouse liver via hydrodynamic tail vein injection at day 40. The livers were analyzed at 24 or 44 weeks of age (n = 9–10 per group). **B** Representative gross morphology of the left lobe of the liver in DEN-treated ZNF498-overexpressing mice at 24 weeks of age. Red arrows indicate visible tumors. **C-E** Total number (**C**), accumulated diameter (**D**) of tumors and the diameter of the largest tumor (E) in each liver of DEN-treated mice at 24 weeks of age. **F** Representative H&E staining of liver tissue sections from DEN-treated control and ZNF498-overexpressing mice. Red arrows indicate examples of nuclear division. **G** Representative gross morphology of the liver in DEN-treated mice at 44 weeks of age. Red arrows indicate visible tumors. **H-J** Total number (**H**), accumulated diameter (**I**) of tumors and the diameter of the largest tumor (**J**) in the livers of DEN-treated mice at 44 weeks of age. **K** Representative histology of the livers in DEN-treated mice at 44 weeks of age. **L** The expression of ZNF498 in liver tumor tissue from DEN-treated mice. Scale bars, 100 μm. *P < 0.05; **P < 0.01; ***P < 0.001

We further explored the role of ZNF498 in the initiation and progression of HCC in a mouse model in which HCC was induced by DEN, a genotoxic hepatocarcinogen [25]. We generated mice with ZNF498 overexpression via hydrodynamic delivery of pLIVE-ZNF498 plasmids. These mice were subjected to DEN treatment starting at 18 days old, and the tumor burden was analyzed at 24 and 44 weeks (Fig. 2A). As indicated in Supplementary Fig. S2A, pLIVE-ZNF498 plasmids injected via the tail vein increased ZNF498 mRNA expression levels in the liver. All mice were alive at 24 and 44 weeks after DEN injection, but compared to control mice, mice with ZNF498 overexpression showed a striking increase in tumor incidence and number of tumors per liver. At 24 weeks, the control mice showed no tumor development, whereas approximately 63.6% of the ZNF498-overexpressing mice presented tumor development (Fig. 2B-E; Supplementary Fig. S2B). Histological analysis of liver sections revealed that ZNF498-overexpressing mice had increased steatosis and nuclear division typically associated with DEN-induced liver damage, indicating HCC initiation [26] (Fig. 2F). At 44 weeks of age, the livers of all control and ZNF498-overexpressing mice were microscopically and macroscopically assessed. Although both sets of mice showed tumor development, ZNF498-overexpressing mice showed significantly more and larger tumors (Fig. 2G-K). ZNF498 protein levels in tumors were higher in pLIVE-ZNF498-injected mice than in control mice, suggesting that ZNF498 overexpression leads to higher tumor numbers and elevates tumor burden in the DEN-induced HCC mouse model (Fig. 2L). Collectively, these findings support the notion that ZNF498 might play an oncogenic role in HCC tumorigenesis and development.

### ZNF498 directly interacts with p53

**Fig. 3:**
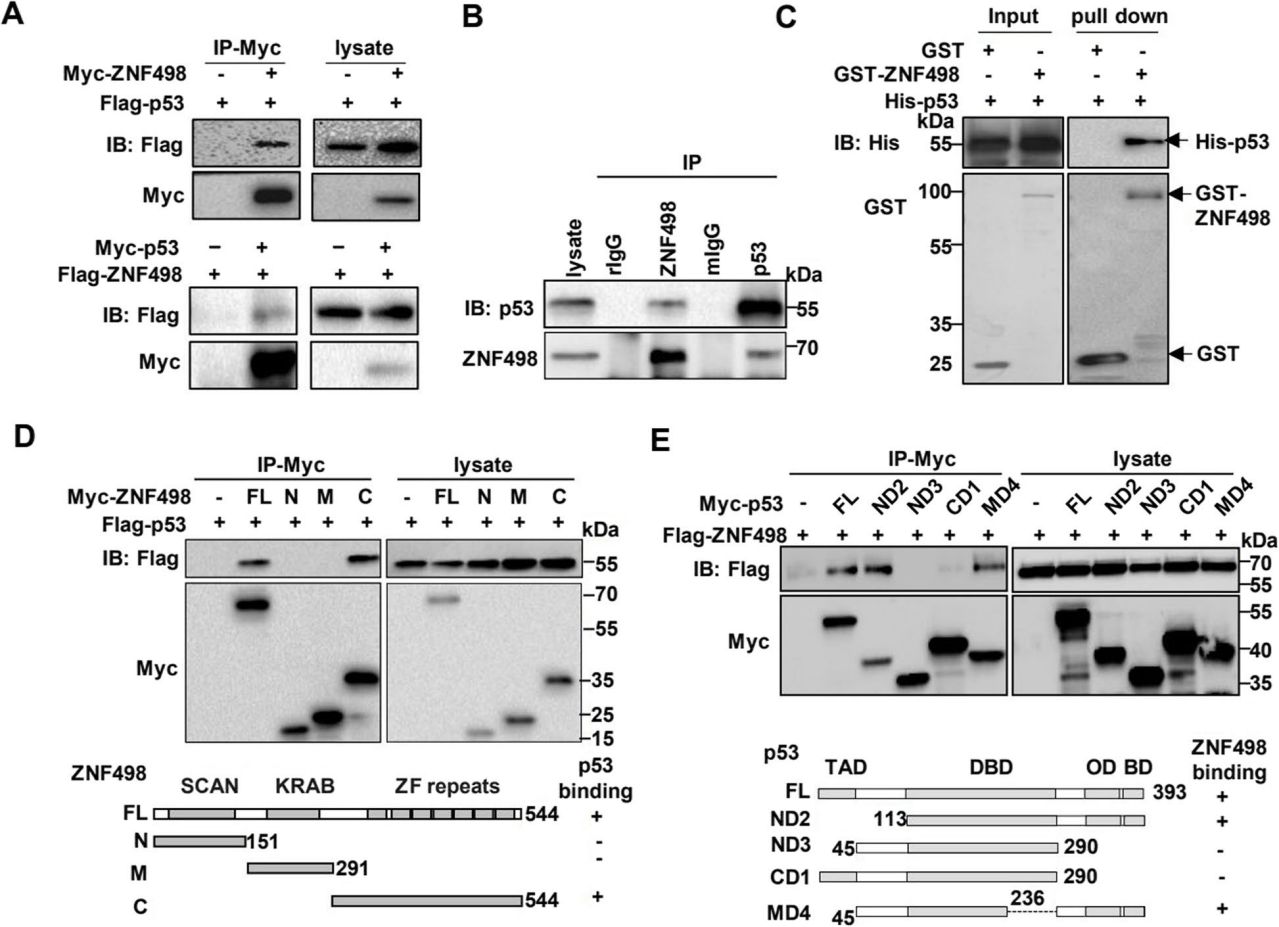
ZNF498 directly interacts with p53 and represses p53 transcriptional activity by inhibiting p53 Ser46 phosphorylation. **A**, **B** Reciprocal co-IP of exogenous (**A**) and endogenous (**B**) ZNF498 and p53 in HepG2 cells. The indicated whole cell lysate and immunoprecipitates were analyzed by Western blotting. rIgG: rabbit IgG, mIgG: mouse IgG. **C** Direct interaction between ZNF498 and p53 as revealed by GST pulldown assays. **D** Map of the region of ZNF498 that interacts with p53. Cell lysates from HCT116 p53^−/−^ cells transfected with Myc-tagged deletion mutants of ZNF498 and Flag-tagged p53 were immunoprecipitated with an anti-Myc antibody before they were subjected to Western blotting. **E** Map of the region of p53 that interacts with ZNF498. Cell lysates from HCT116 p53^−/−^ cells transfected with Flag-tagged ZNF498 and Myc-tagged deletion mutants of p53 were immunoprecipitated with an anti-Myc antibody before they were subjected to Western blotting. FL: full length

Our previous work showed that exogenously expressed ZNF498 could be immunoprecipitated with exogenously expressed p53 [27], suggesting that ZNF498 interacts with p53. Thus, to further explore the mechanism by which ZNF498 affects HCC tumorigenesis and development, we first detected the interaction between ZNF498 and p53 in HepG2 cells. Indeed, exogenous and endogenous ZNF498 and p53 coimmunoprecipitated with each other (Fig. 3A and B). Treatment with the DNA damaging agent etoposide activated p53 but had little effect on the interaction of exogenous and endogenous ZNF498 with p53 (Supplementary Fig. S3A and S3B). To confirm the direct interaction between ZNF498 and p53, we performed GST pulldown assays, and a specific direct interaction between His-p53 and GST-ZNF498 was observed (Fig. 3C). We next used various p53 and ZNF498 deletion mutants to map the domains required for their interaction. Consistent with the results form other members of the KZFP family, Apak (ZNF420), PISA (ZNF568) and PITA (ZNF475) [27, 28], the C-terminus containing zinc fingers (ZFs) (but not the SCAN and KRAB domains) of ZNF498 and the C-terminal residues 291–393 of p53 were required for the interaction between the proteins (Fig. 3 D and E).

### ZNF498 represses p53 transcriptional activity by inhibiting p53 Ser46 phosphorylation

**Fig. 4:**
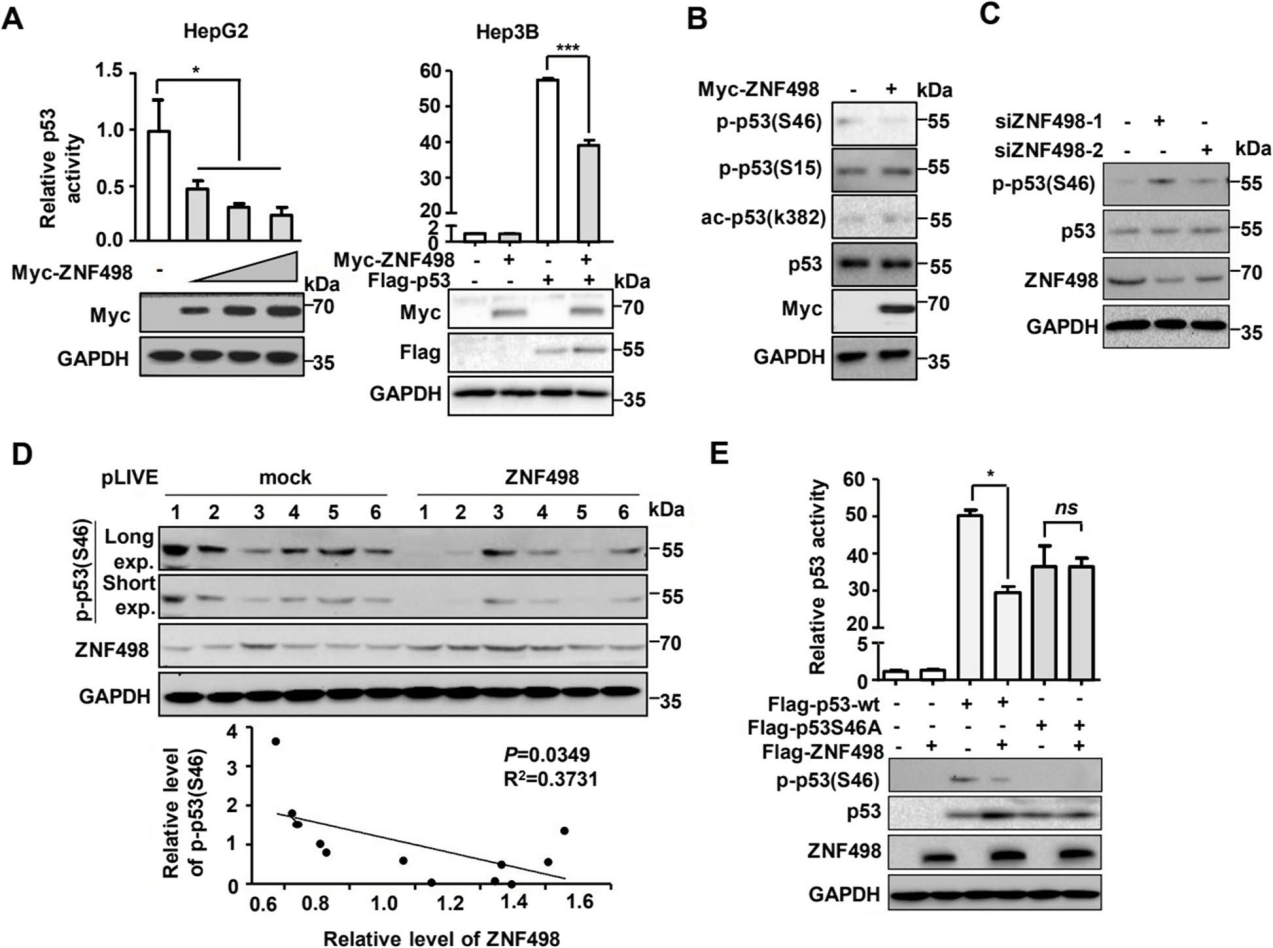
ZNF498 represses p53 transcriptional activity by inhibiting p53 Ser46 phosphorylation. **A** ZNF498 represses p53 transcriptional activity. HepG2 (p53 wild-type) and Hep3B (p53-deficient) cells were transfected with a pG13L reporter plasmid, an internal control reporter plasmid pRL-CMV, and the indicated plasmid. After 36 h of transfection, cells were harvested for dual reporter gene assays (*n* = 3). The expression of ZNF498 and p53 was confirmed by Western blotting. **B** The indicated HepG2 cell lysates were blotted with the indicated antibodies. **C** HepG2 cells transfected with the indicated siRNA were processed for Western blotting. **D** The levels of phosphorylated p53 at Ser46 and ZNF498 in liver tumor tissue from DEN-treated mice were detected and quantified. Regression analysis comparing the protein levels of ZNF498 and phosphorylated p53 at Ser46 is shown below. **E** Hep3B cells were cotransfected with ZNF498 and wild-type p53 or p53S46A, and p53 activity was measured via pG13L luciferase reporter gene assays (n = 3). The expression of ZNF498, p53 and phosphorylated p53 at Ser46 was confirmed by Western blotting. *ns*, no significance; *P < 0.05; **P < 0.01; ***P < 0.001

KZFPs constitute the largest family of transcriptional regulators in mammals and repress transcription by binding to specific DNA sequences or transcription factors. We first investigated whether ZNF498 modulates the transcriptional activity of p53 and observed that ZNF498 significantly repressed the activity of endogenous p53 in a dose-dependent manner in HepG2 cells (p53 wild-type) and that of exogenous p53 in Hep3B cells (p53-null) (Fig. 4A). Even in the presence of etoposide, p53 was activated, and ZNF498 consistently repressed the transactivation activity of p53 (Supplementary Fig. S3C).

We then set out to explore how p53 transcriptional activity is regulated by ZNF498. p53 protein levels showed no significant change in HepG2 control cells verus HepG2 cells with ZNF498 overexpression or knockdown (Fig. 4B and C). However, PTMs of p53 play crucial roles in regulating its activity. Based on our initial results and our knowledge of p53 PTMs, we next examined the effects of ZNF498 on the p53 PTM pattern. Among the various phosphorylation and acetylation sites of p53, Ser46 showed notably decreased phosphorylation in ZNF498-transfected cells whether in the untreated cells or DNA damaging agent-treated groups; however, the phosphorylation or acetylation levels at other residues of p53 remained almost unchanged (Fig. 4B; Supplementary Fig. S3D). Moreover, ZNF498 knockdown resulted in increased p53 phosphorylation at Ser46 (Fig. 4C). Consistently, tissues isolated from tumors of mice with DEN-induced HCC showed a significant negative correlation between ZNF498 levels and p53 Ser46 phosphorylation (Fig. 4D). Next, we separately transfected wild-type p53 and the S46A mutant p53 into Hep3B cells and observed their transcriptional activity in the presence of ZNF498. ZNF498 inhibited wild-type p53 transcriptional activity but had no effect on the transcriptional activity of S46A mutant p53 (Fig. 4E). This further confirms that p53 Ser46 phosphorylation is crucial for ZNF498 to mediate p53 transcriptional activity.

### ZNF498 inhibits p53 phosphorylation at Ser46 by competing with the PKCδ-p53INP1 complex to bind p53

**Fig. 5:**
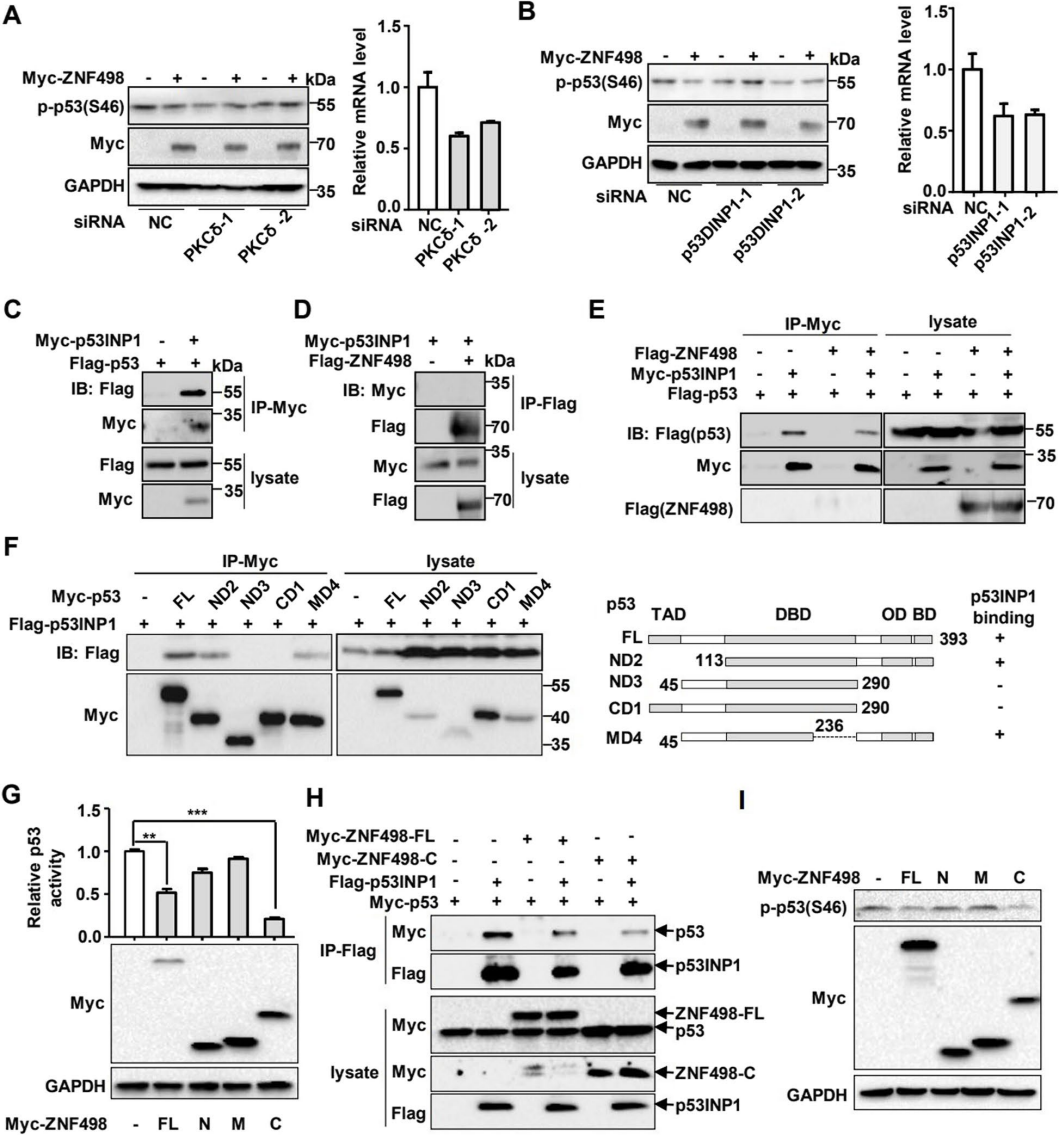
ZNF498 inhibits p53 phosphorylation at Ser46 by competing with the PKCδ-p53INP1 complex to bind p53. **A**, **B** HepG2 cells were transfected with the indicated siRNAs targeting PKCδ (**A**) or p53INP1 (**B**) and analyzed for p53 Ser46 phosphorylation levels by Western blotting. Total RNA from transfected cells was subjected to qPCR using the indicated primers. **C** Immunoprecipitation of exogenous p53INP1 and p53 in HepG2 cells. **D** Immunoprecipitation of exogenous ZNF498 with p53INP1 in 293 T cells. **E** ZNF498 impaired the interaction between p53INP1 and p53. **F** Mapping the region of p53 that interacted with p53INP1. Cell lysates from 293 T cells transfected with Flag-tagged p53INP1 and Myc-tagged deletion mutants of p53 were immunoprecipitated with an anti-Myc antibody and subjected to Western blotting. **G** HepG2 cells were transfected with ZNF498 or its truncated mutants before they were processed for luciferase activity assays. The expression of ZNF498 and its truncated mutants was examined by Western blotting. **H** The C-terminus of ZNF498 containing a ZF domain impaired the interaction between p53INP1 and p53. **I** The C-terminus of ZNF498 downregulated the expression of phosphorylated p53 at Ser46. **P < 0.01; ***P < 0.001

We next explored the molecular mechanism that ZNF498 affects p53 Ser46 phosphorylation. Previous studies have shown that under different cellular stress conditions, p53 is phosphorylated at Ser46 by the kinases PKCδ, HIPK2, DYRK2, ATM and p38α [9]. Knockdown of HIPK2, DYRK2, ATM and p38α showed little impact on the inhibitory effects of ZNF498 on p53 Ser46 phosphorylation (Supplementary Fig. S4A-D), but when PKCδ was knocked down, the inhibitory effect of ZNF498 on the level of p53 Ser46 phosphorylation was significantly abrogated (Fig. 5A). Previous studies have shown that PKCδ associates with p53INP1 (also named p53DINP1) and is recruited to p53 by p53INP1 to phosphorylate p53 at Ser46 [29, 30]. Thus, we explored the role of p53INP1 in the repressive effect of ZNF498 on p53 Ser46 phosphorylation. Knockdown of p53INP1 significantly attenuated the inhibitory effect of ZNF498 on p53 Ser46 phosphorylation (Fig. 5B), suggesting that ZNF498 inhibits p53 Ser46 phosphorylation via PKCδ and the p53INP1 complex.

To investigate the specific mechanism by which ZNF498 inhibits p53 Ser46 phosphorylation via p53INP1 and PKCδ, we examined the associations among ZNF498, p53 and p53INP1. ZNF498 and p53INP1 could interact with p53 but did not interact with each other (Fig. 5C and D; Supplementary Fig. S5). Surprisingly, overexpression of ZNF498 attenuated the interaction between p53 and p53INP1 (Fig. 5E). Similar to ZNF498, p53INP1 interacted with the C-terminus of p53 (Fig. 5F). These results suggested that ZNF498 competes with p53INP1 to bind p53. To confirm this assumption, we examined the effect of the C-terminus of ZNF498, which is responsible for p53 binding. Indeed, the C-terminus of ZNF498 sufficiently suppressed the transcriptional activity of p53, inhibited the interaction between p53 and p53INP1, and downregulated p53 Ser46 phosphorylation (Fig. 5G-I). Taken together, these results demonstrate that ZNF498 downregulates p53 Ser46 phosphorylation by competing with p53INP1 to bind p53.

### ZNF498 functions as an oncogene in a p53-dependent manner

**Fig. 6:**
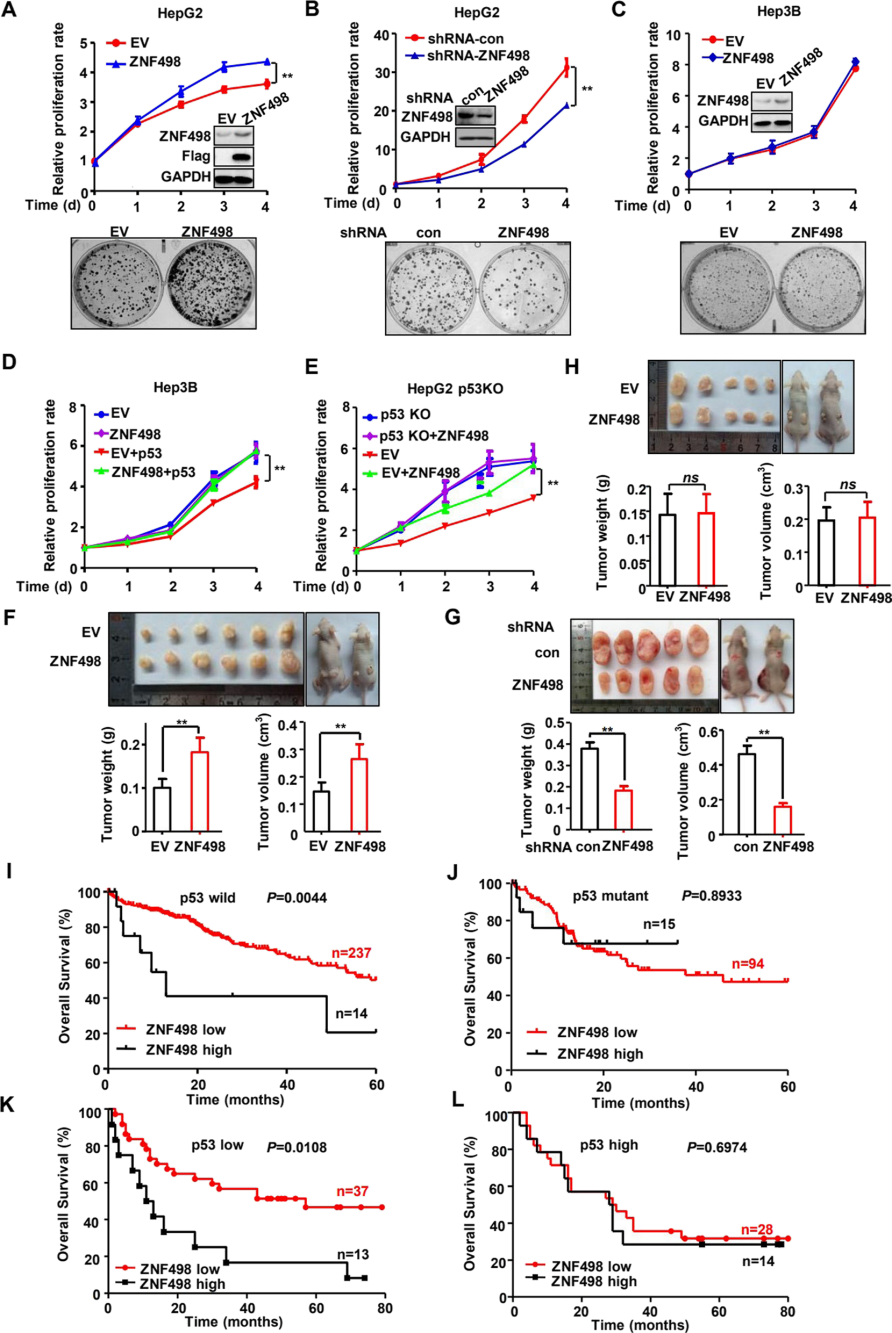
ZNF498 functions as an oncogene in a p53-dependent manner. **A, B, C** HepG2 cells with stable overexpression (**A**) or knockdown (**B**) of ZNF498 and Hep3B cells with overexpression of ZNF498 (**C**) were cultured for CCK-8 and colony formation assays. ZNF498 expression was detected using Western blotting. **D** p53 reintroduction restored the promoting effect of ZNF498 on the growth of Hep3B cells. EV and ZNF498-overexpressing Hep3B cells were transfected with or without the p53 expression vector, and cell proliferation was measured using a CCK-8 assay. **E** p53 knockout diminished the promoting effect of ZNF498 on the growth of HepG2 cells. EV and p53-knockout HepG2 cells were transfected with or without the ZNF498 expression vector, and cell proliferation was measured using the CCK-8 assay. **F-H** A total of 1.5 × 10^6^ HepG2 cells with or without overexpression of ZNF498 (**F**), 4 × 10^6^ HepG2 cells with or without downregulation of ZNF498 (**G**), and 1.5 × 10^6^ p53- knockout HepG2 cells with or without overexpression of ZNF498 (**H**) were injected into nude mice, which were then housed for different times. Tumors were isolated, and tumor weight and volume were measured. **I, J** The association between ZNF498 mRNA levels and OS in TP53 WT and TP53 mutated subgroups of patients with HCC was evaluated by Kaplan–Meier analysis. **K, L** Kaplan–Meier survival curves for p53-low and p53-high HCC patients, respectively. *ns*: no significance; **P < 0.01

We next investigated whether ZNF498 plays an oncogenic role in a p53-dependent manner. As ZNF498 is widely expressed in various liver cancer cell lines (Supplementary Fig. S6A), we assessed the effects of ZNF498 on their growth. ZNF498 overexpression in HepG2 cells dramatically increased cell growth, as detected by CCK-8 and colony formation assays (Fig. 6A). Conversely, knockdown of ZNF498 in HepG2 cells significantly suppressed cell growth and clonogenicity (Fig. 6B). Consistently, ZNF498 overexpression dramatically increased the proliferation of L-02 and SMMC7721 cells expressing wild-type p53 (Supplementary Fig. S6B and C). However, ZNF498 overexpression had no effect on the growth of p53-null Hep3B cells (Fig. 6C). Ectopic expression of p53 inhibited cell growth and rescued the growth-promoting effect of ZNF498 in Hep3B cells (Fig. 6D). To further confirm that the oncogenic role of ZNF498 is dependent on p53, p53-knockout HepG2 cells were established (Supplementary Fig. S7); these cells did not exhibit accelerated cell growth when ZNF498 was overexpressed, and ectopic expression of p53 rescued the cell growth-promoting effect of ZNF498 (Fig. 6E).

We next evaluated the function of ZNF498 on in vivo cell growth using a xenograft mouse model. Compared to those derived from control cells, tumors derived from HepG2 cells with ZNF498 overexpression had a significantly higher tumor weight and volume in mice (Fig. 6F), whereas tumors derived from HepG2 cells with ZNF498 knockdown presented significantly less tumor growth (Fig. 6G). Furthermore, tumor growth was not difference between nude mice xenografted with ZNF498-overexpression p53-knockout HepG2 cells versus p53-knockout cells (Fig. 6H). Collectively, these results demonstrate that ZNF498 promotes HCC cell growth in vitro and in vivo in a p53-dependent manner.

To verify that ZNF498 exerts effects on HCC in a p53-dependent manner in clinical samples, we collected *TP53* mutational information from the TCGA HCC cohort and found that the correlation between ZNF498 expression and patient outcome was significant only in those with wild-type *TP53* (Fig. 6I and J). We further conducted IHC staining to analyze the protein expression of ZNF498 and p53. In many tumors, including HCC, increased p53 levels are a result of p53 mutation, and IHC for p53 is an acceptable surrogate test for TP53 mutational analysis. According to the IHC staining scores for p53, HCC patients were divided into 2 groups: p53-high (presumably p53-mutant) and p53-low (presumably p53-wild type) [31, 32]. Upregulated expression of p53 was detected in 42 of 92 (45.65%) tumor tissues, which is consistent with the mutation rate of p53 stated in previous reports [33]. ZNF498 expression was significantly upregulated in HCC cancer tissues compared with adjacent normal liver tissues, but the statistical differences in the p53-low group were significantly higher than those in the p53-high group (Supplementary Fig. S8A and B). Furthermore, when only p53-low cases were considered, high ZNF498 expression was significantly correlated with histological grade (Supplementary Fig. S8C and D). Patients with high ZNF498 expression showed worse survival than those with low ZNF498 expression; this correlation was not observed in the p53-high HCC cases (Fig. 6K and L). Taken together, these findings further support that ZNF498 promotes HCC carcinogenesis in a p53-dependent manner.

### ZNF498 suppresses apoptosis and ferroptosis via the regulation of p53 Ser46 phosphorylation-mediated p53 transcriptional activity in HCC cells

**Fig. 7:**
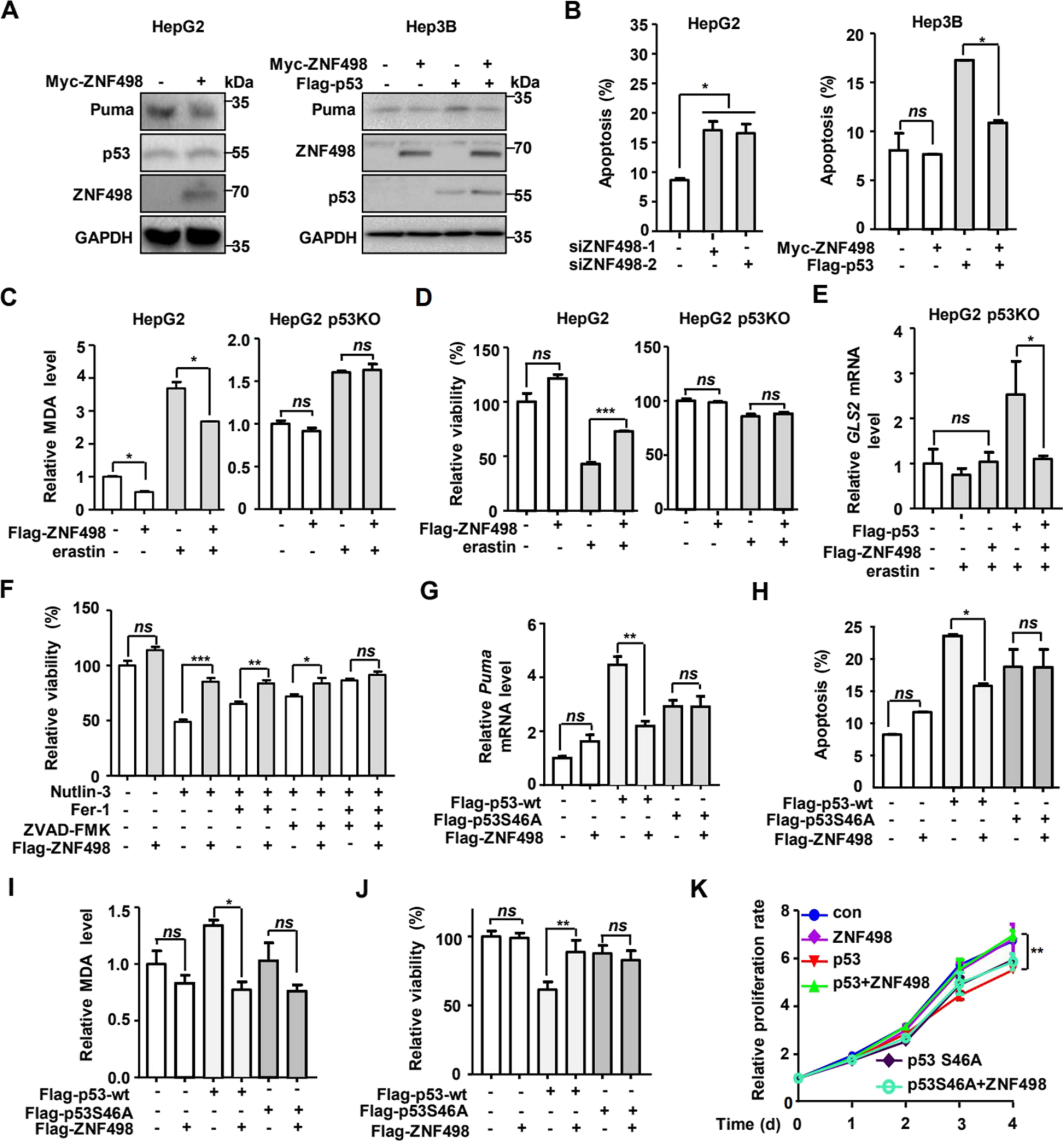
ZNF498 suppresses apoptosis and ferroptosis via p53 Ser46 phosphorylation-mediated p53 transcriptional activation. **A** HepG2 and Hep3B cells were transfected with the indicated plasmids for 36 h and collected for Western blotting. **B** HepG2 and Hep3B cells were transfected with the indicated siRNA or plasmids for 36 h and collected for apoptosis detection. **C** HepG2 and p53-knockout HepG2 cells were transfected with ZNF498 expression plasmid and treated with or without erastin (10 μM) for 24 h. The level of MDA was measured using a lipid peroxidation assay kit. **D** Cell viability was determined by CCK-8 assays in HepG2 and p53-knockout HepG2 cells transfected with ZNF498 expression plasmid and treated with or without erastin (40 μM) for 48 h. **E** p53-knockout HepG2 cells were transfected and treated as indicated, and *GLS2* mRNA levels were analyzed by qPCR. **F** HepG2 cells were transfected as indicated and treated with Nutlin-3 (20 μmol/L) with or without cell death inhibitors (Fer-1, 2 μmol/L; ZVAD-FMK, 2 μmol/L) for 24 h, and cell viability was assayed. **G** Hep3B cells were transfected with plasmids as indicated for 48 h and subjected to qPCR to determine *Puma* mRNA levels. **H** Hep3B cells were transfected with the indicated plasmids and collected for apoptosis detection. **I, J** p53-knockout HepG2 cells were transfected with the indicated plasmids, treated with erastin (10 μM) for 24 h, and processed to detect MDA levels (**I**) and cell viability (**J**). **K** p53-knockout HepG2 cells were transfected with the indicated plasmids, and cell proliferation was measured using a CCK-8 assay. *n* = 3; *ns*, no significance; *P < 0.05; **P < 0.01

p53 Ser46 phosphorylation occurs in response to severe genotoxicity and many kinds of cellular stress and activates cell death via apoptosis and ferroptosis pathways [9, 10]. Therefore, on the basis of our results, we speculated that ZNF498 promotes apoptosis and ferroptosis via regulation of p53 Ser46 phosphorylation-mediated p53 transcriptional activity in HCC cells. We first examined the ability of ZNF498 to regulate the transcription of p53 target genes. In HepG2 cells, ZNF498 overexpression significantly reduced the mRNA levels of the proapoptotic genes *Puma* and *p53AIP1*, but had no or weak effects on the antiapoptotic genes *p21* and *14–3-3σ;* the DNA repair genes *p53R2*, *DDB1* and *XPC;* the senescence-related genes *PML* and *PAI;* the drug resistance gene *MDR;* the self-regulatory gene *HDM2;* and the negative regulatory gene *cdc25c* (Supplementary Fig. S9A). Consistently, when ZNF498 was knocked down in HepG2 cells, the mRNA levels of *Puma* and *p53AIP1* were increased. ZNF498 overexpression attenuated the p53-induced increases in *Puma* and *p53AIP1* mRNA expression in Hep3B cells (Supplementary Fig. S9B and C). Accordingly, the ZNF498-induced changes in Puma expression were consistent at the mRNA and protein levels (Fig. 7A and B; Supplementary Fig. S9D). We also detected the effect of ZNF498 on other proapoptotic target genes of p53, *Bax* and *Noxa*. Similar to the results for Puma, ZNF498 significantly decreased the mRNA levels of *Bax* and *Noxa* (Supplementary Fig. S9E). Considering the proapoptotic role of these genes, we next examined whether ZNF498 affects apoptosis. ZNF498 knockdown significantly increased the apoptosis rate of HepG2 cells, whereas ZNF498 overexpression significantly suppressed p53-induced apoptosis in Hep3B cells (Fig. 7B). TUNEL assays also confirmed that ZNF498 overexpression reduced apoptosis in a p53-dependent manner (Supplementary Fig. S9F). In cells treated with the DNA damaging agent etoposide, ZNF498 consistently downregulated Puma protein levels and inhibited p53-mediated apoptosis (Supplementary Fig. S9G and H). Taken together, these results suggest that ZNF498 is a negative regulator of p53 and inhibits p53-mediated apoptosis by repressing the expression of the proapoptotic target genes of p53.

We further examined whether ZNF498 influences the cellular sensitivity to ferroptosis. Ferroptosis is an iron-dependent, oxidative form of regulated cell death and is characterized by reduced intracellular GSH levels, elevated ROS levels, and the accumulation of lipid peroxides during cell death [34, 35]. In HepG2 cells treated with erastin, a classical ferroptosis inducer [36], ZNF498 overexpression reduced the levels of MDA, one of the end products of lipid peroxidation [34, 37], and ROS, increased GSH levels and promoted cell survival (Fig. 7C and D left; Supplementary Fig. S10A and S10B). To confirm the repressive effect of ZNF498 on ferroptosis, we further assessed the effect of ZNF498 on cell survival and GSH and ROS levels under treatment with two other ferroptosis inducers, RSL-3 [38] and IKE [39]. Consistently, ZNF498 reduced RSL-3- and IKE-induced ROS upregulation, GSH downregulation and cell death in HepG2 cells (Supplementary Fig. S10C-E). Furthermore, ZNF498 promoted cell viability induced by erastin, which could be blocked partially by the ferroptosis inhibitor Fer-1 (Supplementary Fig. S10F). Previous research has revealed that p53 promotes ferroptosis by regulating the expression of GLS2 [10, 40], SLC7A11 [36], ALOX12 [41], PTGS2 [42] and SAT1 [43]. We detected the effect of ZNF498 on the expression of the pro-ferroptosis target genes of p53. Interestingly, we found that ZNF498 specifically decreased the mRNA level of GLS2 in HepG2 cells and had no effect on the other pro-ferroptosis p53 targets (Fig. 7E; Supplementary S10G). All of the above results confirmed that ZNF498 could inhibit ferroptosis.

We further examined whether ZNF498 regulates ferroptosis in a p53-dependent manner. ZNF498 showed little effect on p53-knockout HepG2 cells, which is inconsistent with its effect in HepG2 cells (Fig. 7C and D). Furthermore, in erastin-treated p53-knockout HepG2 cells, ZNF498 inhibited p53-induced expression of the target gene *GLS2* (Fig. 7E). These results indicate that the inhibitory effect of ZNF498 on ferroptosis is p53 dependent.

Apoptosis and ferroptosis are two different forms of programmed cell death that play vital roles in p53-mediated tumor suppression. We next explored the correlation between apoptosis and ferroptosis in p53-mediated cell death by using Nutlin-3 to activate p53. Both Fer-1 and the apoptosis inhibitor ZVAD-FMK partially but significantly attenuated Nutlin-3-mediated cell death, indicating that p53 could induce apoptosis and ferroptosis in HepG2 cells. In addition, ZNF498 promoted cell viability induced by Nutlin-3, which could be partially blocked by Fer-1 and ZVAD-FMK. The combination of ferrostatin-1 and ZVAD-FMK fully blocked the promoting effect of ZNF498 on cell viability (Fig. 7F). These results indicated that ZNF498 simultaneously suppresses p53-mediated apoptosis and ferroptosis in HepG2 cells.

Considering the inhibitory effect of ZNF498 on the phosphorylation of p53 Ser46, we assessed whether the inhibitory effects of ZNF498 on apoptosis and ferroptosis in HCC cells were dependent on p53 Ser46 phosphorylation. The inhibitory effects of ZNF498 on Puma expression and apoptosis were prevalent in Hep3B cells transfected with wild-type p53 but not in the Hep3B cells transfected with p53 S46A mutant p53 (Fig. 7G; Supplementary S11A). Similar results regarding the inhibitory effects of ZNF498 on Puma expression and apoptosis were obtained in p53-deficient HCT116 cells (Supplementary Fig. S11B-E). We also performed rescue experiments in p53-knockout HepG2 cells and found that overexpression of wild-type p53, but not its S46A mutant, enabled ZNF498 to inhibit ferroptosis in the presence of erastin (Fig. 7 I and J). Moreover, the growth-suppressive effects of ZNF498 were restored in p53-knockout HepG2 cells with ectopic wild-type p53 expression but not with S46A mutant p53 expression (Fig. 7K). Hence, these data imply that p53 Ser46 phosphorylation is crucial for the tumor-promoting effects of ZNF498.

## Discussion

**Fig. 8:**
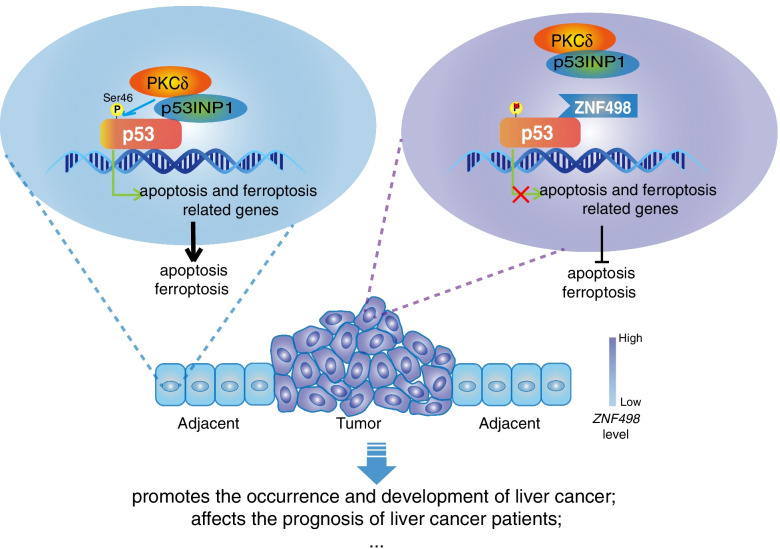
Simplified schematic diagram indicating the potential role of ZNF498 in promoting the tumorigenesis and progression of HCC

HCC is a kind of malignant tumor with an unsatisfactory prognosis. Abnormal gene expression is significantly associated with the initiation and poor prognosis of HCC. Here, we found that ZNF498 is overexpressed in human HCC tissues and competes with p53INP1 for p53 binding. The dissociation of p53INP from p53 attenuates PKCδ-mediated p53 Ser46 phosphorylation, which suppresses p53 transcriptional activity, downregulates proapoptotic and ferroptotic target genes of p53, inhibits p53-mediated apoptosis and ferroptosis, and promotes HCC initiation and progression (Fig. 8).

ZNF498 is a member of the KZFP family, the largest family of transcriptional regulators found in higher vertebrates. Previous reports have revealed that the majority of KZFPs identified in humans bind TEs, control TE activity and the expression of nearby genes during the early stages of embryonic development and in some adult tissues, and play key roles in early development and many physiological events [15, 44–46]. However, recent reports have revealed that some members of this large family might have acquired new roles, especially with regard to tumorigenesis and progression, that occur in a TE-binding-independent manner [16, 20, 47]. Our work confirmed that ZNF498 can directly interact with p53, decrease p53 Ser46 phosphorylation and inhibit p53 transcriptional activity. p53 Ser46 phosphorylation can lead to the separation of p53 from the antiapoptotic iASPP protein and the binding of p53 to the prolyl-peptidyl cis/trans isomerase Pin1, which catalyzes isomerization of the phospho-Ser46-Pro47 bond, allowing the interaction of p53 with the acetyltransferases CBP and p300; thus results in the acetylation of p53 and induces the expression of apoptotic p53 target genes [9, 48]. Moreover, p53 phospho-Ser46 has been implicated in regulating ferroptosis by modulating the expression of GLS2 and SLC7A11, which are essential for ferroptosis and exhibit tumor suppressor activities in human HCC [40, 49]. Interestingly, ZNF498 specifically inhibits the p53 pro-ferroptosis target gene GLS2 and widely suppresses p53 apoptotic target genes. In addition to regulating p53 Ser46 phosphorylation, other mechanisms may be involved in ZNF498 regulation of p53 progression and ferroptosis target gene expression, and these mechanisms should be explored in future work. The roles of p53 as a critical tumor suppressor are well established: it can regulate cell cycle arrest, apoptosis, senescence, and especially metabolic and ferroptosis regulation; these activities are critical for tumor suppression. As a negative regulator of p53 Ser46 phosphorylation, ZNF498 inhibits p53-mediated apoptosis and ferroptosis and promotes HCC cell growth in vivo and in vitroin a p53-dependent manner. Taken together, our findings shed light on the critical role of ZNF498 as a novel repressor of p53 in HCC initiation and development.

The KRAB domain of KZFPs plays an essential role in the transcriptional repression of KZFPs; this domain typically recruits the corepressor KRAB-associated protein 1 (KAP1, also known as TRIM28 and Tif1β). This protein serves as a scaffold for the further recruitment of corepressors, such as HP1, SETDB1, and NuRD complexes, and the recruitment of DNMTs. The C2H2 ZF domains determine the DNA-binding specificity of KZFP [11, 50]. However, the suppression of p53 transcriptional activity by ZNF498 is independent of its KRAB domain and the DNA-binding ability of its ZFs. Both ZNF498 and p53INP1, a regulator of p53 phosphorylation at Ser46, interact with the C-terminus of p53. ZNF498 inhibits the interaction between p53 and p53INP1, deregulates the activation of PKCδ (which is recruited to p53 by p53INP1), and consequently attenuates p53 Ser46 phosphorylation. Our previous study also showed that other members of the KZFP family are involved in p53 regulation. Apak specifically dampens p53-mediated apoptosis, whereas PISA and PITA are selective regulators of p53 in metabolic control. These KZFPs regulate p53 activity via different molecular mechanisms [27, 28]. Apak quenches p53 acetylation, competitively hinders the binding of p53 to the p53 responsive element (RE) of the proapoptotic gene *p53AIP1*, and negatively regulates p53 by two complementary, class-specific and gene-specific molecular mechanisms. PITA selectively inhibits p53 binding to the *TIGAR* gene, and PISA selectively inhibits p53 binding to the *SCO2* gene by directly competing with p53 binding to the *SCO2* gene. The function and mechanism of ZNF498 in p53 regulation are different from those of the other three KZFPs. ZNF498 competes with p53INP1 to bind p53 and attenuates p53 Ser46 phosphorylation.

p53 is a key signaling node in the response to diverse cellular stresses, and DNA damage or cellular stress can lead to the dissociation of many kinds of p53 repressors from p53, resulting in p53 activation to suppress tumor initiation and progression. Under different stress conditions, p53 regulators in the KZFP family dissociate from p53. For example, DNA damage and glucose starvation results in the dissociation of Apak, PISA and PITA from p53 [27, 28]. However, DNA damage has little effect on the interaction of ZNF498 with p53, and ZNF498 suppresses p53 transcriptional activity, p53 Ser46 phosphorylation and p53-induced apoptosis and ferroptosis. Sustained inhibition of p53 by ZNF498 is essential for its promoting effects on HCC initiation and progression.

## Conclusion

In summary, we demonstrated that ZNF498 plays a crucial role in promoting HCC carcinogenesis by suppressing apoptosis and ferroptosis via its interaction with p53 to downregulate p53 Ser46 phosphorylation. These findings highlight the importance of the ZNF498-p53 signaling axis in the control of hepatocellular carcinogenesis and could enrich our understanding of p53-inactivating mechanisms that occur independent of mutations in HCC and of the regulatory mechanisms of the KZFP family. Moreover, our results suggest that ZNF498 is a potential biomarker and new therapeutic target for HCC.

## Supplementary Information

**Additional file 1: Supplementary Figure S1.** Anti-ZNF498 antibody specifically recognizes ZNF498. **Supplementary Figure S2.** ZNF498 promotes the initiation of DEN-induced HCC. **Supplementary Figure S3.** ZNF498 interacts with p53, represses p53 transcriptional activity and inhibits p53 Ser46 phosphorylation under DNA damage conditions. **Supplementary Figure S4.** ZNF498 has no effect on p53 Ser46 phosphorylation in HCC cells with knockdown of DYRK2, ATM, HIPK1 and p38. **Supplementary Figure S5.** ZNF498 does not interact with p53INP1. **Supplementary Figure S6.** ZNF498 promotes HCC cell growth *in vitro*. **Supplementary Figure S7.** p53 expression was identified in HepG2 cells with stable knockout of p53. **Supplementary Figure S8.** The correlation between ZNF498 overexpression and different p53 statuses in HCC tissues. **Supplementary Figure S9.** ZNF498 represses p53-mediated apoptosis. **Supplementary Figure S10.** ZNF498 represses ferroptosis. **Supplementary Figure S11.** ZNF498 represses p53 activity and apoptosis by inhibiting p53 Ser46 phosphorylation.

**Additional file 2: Table S1.** Antibodies information.

**Additional file 3: Table S2.** siRNA sequences.

**Additional file 4: Table S3. **ZNF498 mRNA level and *TP53* mutational status in HCC patients from TCGA datasets.

**Additional file 5: Table S4.** Primer sequences.

**Additional file 6: Table S5.** Correlation between expression levels of ZNF498 and clinicopathological characteristics in patients with HCC.

## Data Availability

All data generated or analyzed during this study are included in this published article.

## Abbreviations

HCC: Hepatocellular carcinoma
PTM: Post-translational modification
Ser: Serine
KRAB: Krüppel-associated box
KZFPs: Krüppel-associated box domain zinc finger proteins
ZF: Zinc finger
TEs: Transposable elements
Fer-1: Ferrostatin-1
TCGA: The Cancer Genome Atlas
DEN: Diethylnitrosamine
IHC: Immunohistochemistry
CDS: Coding sequence
TMAs: Tissue microarrays
Co-IP: Co-immunoprecipitation
GST: Glutathione S-transferase
WT: Wild-type
KAP1: KRAB-associated protein 1
p53 KO: p53 knockout
MDA: Malondialdehyde
GSH: Glutathione
ROS: Reactive oxygen species
RE: Responsive element
