# Supplementary material for: ZNF498 promotes hepatocellular carcinogenesis by suppressing p53-mediated apoptosis and ferroptosis via the attenuation of p53 Ser46 phosphorylation
Source: J Exp Clin Cancer Res. 2022 Feb 28;41:79. doi: 10.1186/s13046-022-02288-3 (PMC8883630; doi:10.1186/s13046-022-02288-3)
Supplement: Supplementary file 1 — Additional file 1: Supplementary Figure S1. Anti-ZNF498 antibody specifically recognizes ZNF498. Supplementary Figure S2. ZNF498 promotes the initiation of DEN-induced HCC. Supplementary Figure S3. ZNF498 interacts with p53, represses p53 transcriptional activity and inhibits p53 Ser46 phosphorylation under DNA damage conditions. Supplementary Figure S4. ZNF498 has no effect on p53 Ser46 phosphorylation in HCC cells with knockdown of DYRK2, ATM, HIPK1 and p38. Supplementary Figure S5. ZNF498 does not interact with p53INP1. Supplementary Figure S6. ZNF498 promotes HCC cell growth in vitro. Supplementary Figure S7. p53 expression was identified in HepG2 cells with stable knockout of p53. Supplementary Figure S8. The correlation between ZNF498 overexpression and different p53 statuses in HCC tissues. Supplementary Figure S9. ZNF498 represses p53-mediated apoptosis. Supplementary Figure S10. ZNF498 represses ferroptosis. Supplementary Figure S11. ZNF498 represses p53 activity and apoptosis by inhibiting p53 Ser46 phosphorylation. [file 13046_2022_2288_MOESM1_ESM.zip › Supplementary information-ZNF498-JECCR.docx]

**ZNF498 promotes hepatocellular carcinogenesis by suppressing p53-mediated apoptosis and ferroptosis via the attenuation of p53 Ser46 phosphorylation**

(Supplementary Information)

**SUPPLEMENTARY FIGURE LEGENDS**

**Supplementary Figure S1.** Anti-ZNF498 antibody specifically recognizes ZNF498. **A** Western blotting analysis for anti-ZNF498 immunoprecipitate and whole cell lysate from HepG2 cells transfected with Myc-ZNF498. **B** Immunofluorescence analysis with anti-ZNF498 antibody was performed. The nuclei were stained with DAPI. Images were captured by confocal microscopy and merged. Scale bar, 10 μm. **C** HepG2 cells transfected with the indicated siRNA were collected for Western blotting using anti-ZNF498 antibody.

**Supplementary Figure S2.** ZNF498 promotes the initiation of DEN-induced HCC. **A** ZNF498 mRNA levels in the livers of control and ZNF498-overexpressing mice were detected using qPCR. **B** Number of mice with and without visible tumors (≥1 mm) in the liver. ****P* < 0.001.

**Supplementary Figure S3.** ZNF498 interacts with p53, represses p53 transcriptional activity and inhibits p53 Ser46 phosphorylation under DNA damage conditions. **A** Co-IP of exogenous ZNF498 and p53 in the presence of etoposide. **B** Co-IP of endogenous ZNF498 and p53 in the presence of etoposide. After HepG2 cells were incubated with or without etoposide, co-IP assays were performed using the indicated antibody, and the immunoprecipitates were blotted. **C** ZNF498 represses p53 transcriptional activity under etoposide treatment. After 24 h of transfection, HepG2 cells were incubated with or without etoposide (100 μM) for 18 h. The expression of ZNF498 and p53 was confirmed by Western blotting and is shown at the bottom. **D** The effects of ZNF498 on PTM of p53. After 24 h of transfection, HepG2 cells were incubated with or without cisplatin (20 μM), etoposide (100 μM) for 18 h. The expression of PTM of p53, ZNF498 and p53 was confirmed by Western blotting. **P* < 0.05, unpaired Student’s t test.

**Supplementary Figure S4.** ZNF498 has no effect on p53 Ser46 phosphorylation in HCC cells with knockdown of DYRK2, ATM, HIPK1 and p38. HepG2 cells were transfected with the indicated siRNAs targeting (**a**) DYRK2, (**b**) ATM, (**c**) HIPK1 and (**d**) p38. Total RNA and cell lysates from transfected cells were subjected to qPCR and Western blotting using the indicated primers and antibodies.

**Supplementary Figure S5.** ZNF498 does not interact with p53INP1. Co-IP of exogenous ZNF498 with p53INP1 in 293T cells.

**Supplementary Figure S6.** ZNF498 promotes HCC cell growth *in vitro*. **A** The protein level of ZNF498 in a panel of HCC cell lines. **B, C** Overexpression of ZNF498 promotes the proliferation of L-02 (**B**) and SMMC7721 cells (**C**). Left, confirmation of ZNF498 overexpression in L-02 and SMMC7721 cells. **P* < 0.05, ***P* < 0.01.

**Supplementary Figure** **S7.** p53 expression was identified in HepG2 cells with stable knockout of p53. HepG2 cells with stable knockout of p53 were established by lentivirus transduction, and p53 expression was detected using Western blotting.

**Supplementary Figure S8.** The correlation between ZNF498 overexpression and different p53 statuses in HCC tissues. **A, B** ZNF498 expression in 47 pairs of **(A)** p53-low (scores<5) and **(B)** 39 matched pairs of p53-high (scores≥5) HCC tissues and their matched adjacent normal tissues was assessed using IHC. Representative images are shown. **C, D** Statistical analysis and representative images of ZNF498 expression in p53-low and p53-high tumors with different histological grades.

**Supplementary Figure S9.** ZNF498 represses p53-mediated apoptosis. **A** Total RNA from transfected HepG2 cells was subjected to qPCR. **B** ZNF498 knockdown increased the levels of the apoptosis-related p53 target genes *Puma* and *p53AIP1*. HepG2 cells transfected with the indicated siRNA were collected for qPCR. **c** In Hep3B cells, ZNF498 decreased the mRNA levels of the apoptosis-related p53 target genes *Puma* and *p53AIP1* in a p53-dependent manner. **D** Knockdown of ZNF498 increased the protein level of Puma. HepG2 cells transfected with the indicated siRNA were collected for Western blotting. **E** ZNF498 decreased the mRNA levels of the apoptosis-related p53 target genes *Bax* and *Noxa.* **F** p53-knockout HepG2 cells were transfected as indicated, and the rate of apoptosis was examined using the TUNEL assay. **G** ZNF498 decreased Puma protein levels under etoposide treatment conditions. HepG2 cells transfected with the indicated plasmids or siRNA were treated with etoposide and collected for Western blotting. **H** HepG2 cells transfected with the indicated siRNA were treated with or without etoposide and collected for Annexin V/propidium iodide (PI) staining. **P* < 0.05; ***P* < 0.01.

**Supplementary Figure S10.** ZNF498 represses ferroptosis. **A** ROS levels were measured in HepG2 cells transfected with ZNF498 expression plasmid and treated with or without erastin (10 μM) for 24 h. **B** GSH levels were measured in HepG2 cells transfected with ZNF498 expression plasmid and treated with or without erastin (10 μM) for 24 h. **C** ROS levels were measured in HepG2 cells transfected with ZNF498 expression plasmid and treated with or without IKE (2 μM) and RSL3 (2 μM) for 24 h. **D** GSH levels were measured in ZNF498 transfected-HepG2 cells treated with or without IKE (2 μM) and RSL3 (2 μM) for 24 h. **E** Cell viability was determined by CCK-8 assays in ZNF498 transfected-HepG2 cells treated with or without IKE (2 μM) and RSL3 (2 μM) for 48 h. **F** Cell viability was determined by CCK-8 assays in ZNF498 transfected-HepG2 cells treated with or without erastin (10 μM) and inhibitors (Fer-1, 2 μmol/L) for 48 h. **G** HepG2 cells were transfected as indicated and treated with or without erastin (10 μM) for 24 h. Total RNA was subjected to qPCR. *ns*, no significance, **P* < 0.05; ***P* < 0.01.

**Supplementary Figure S11.** ZNF498 represses p53 activity and apoptosis by inhibiting p53 Ser46 phosphorylation. **A** ZNF498 decreased Puma protein levels based on the phosphorylation of p53 at Ser46. Hep3B cells were transfected with plasmids as indicated for 48 h and subsequently harvested for Western blotting. **B** The repressive effects of ZNF498 on p53 activity depended on p53 Ser46 phosphorylation in HCT116 p53^-/-^ cells. **C** ZNF498 repressed Puma gene transcription in a p53 Ser46 phosphorylation-dependent manner in HCT116 p53^-/-^ cells. **D** ZNF498 decreased Puma protein levels in a p53 Ser46 phosphorylation-dependent matter in HCT116 p53^-/-^ cells. **E** The inhibitory effect of ZNF498 on p53-mediated apoptosis depended on p53 Ser46 phosphorylation in HCT116 p53^-/-^ cells. *ns*, no significance, ***P* < 0.01.
