# Supplementary figures and images for: ZNF498 promotes hepatocellular carcinogenesis by suppressing p53-mediated apoptosis and ferroptosis via the attenuation of p53 Ser46 phosphorylation
Source: J Exp Clin Cancer Res. 2022 Feb 28;41:79. doi: 10.1186/s13046-022-02288-3 (PMC8883630; doi:10.1186/s13046-022-02288-3)

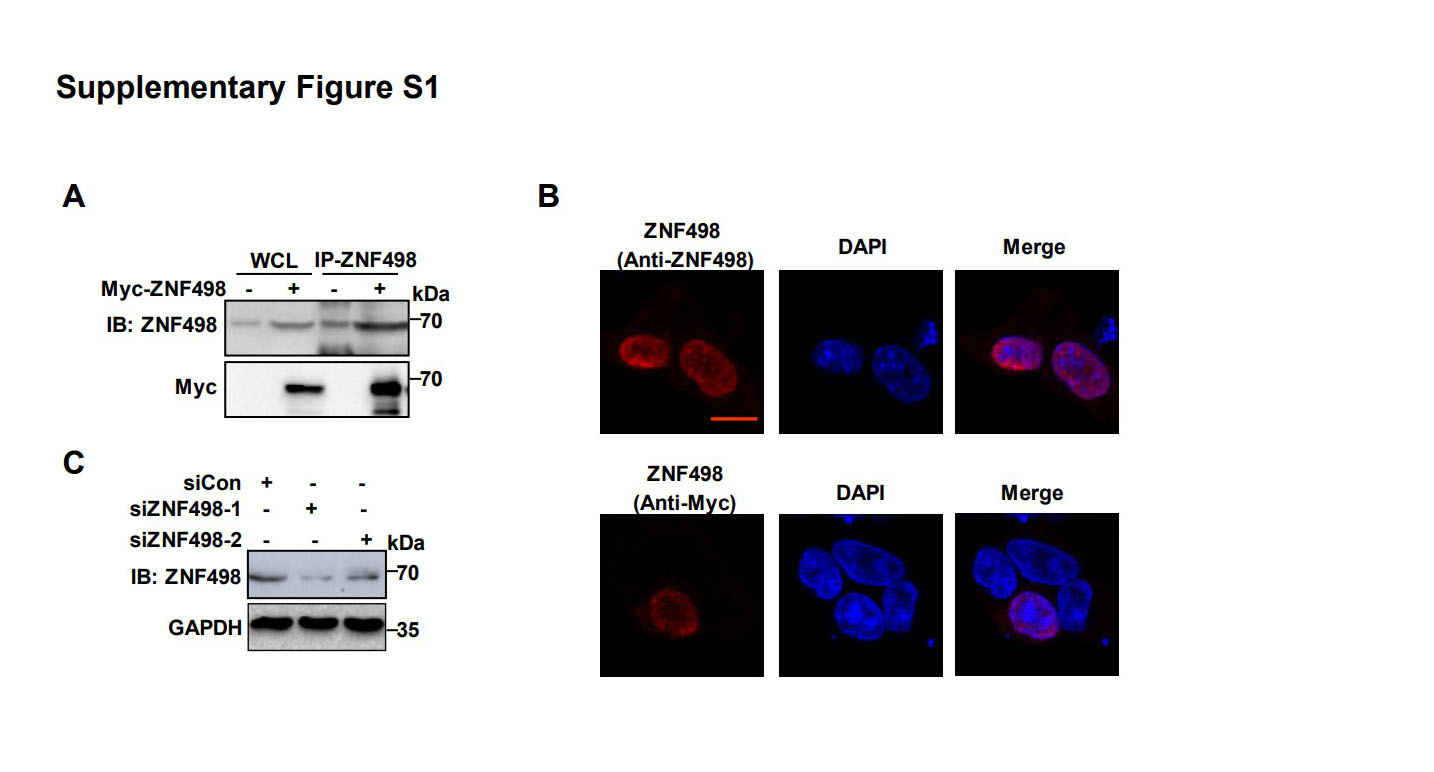

Supplement: Supplementary file 1 — Additional file 1: Supplementary Figure S1. Anti-ZNF498 antibody specifically recognizes ZNF498. Supplementary Figure S2. ZNF498 promotes the initiation of DEN-induced HCC. Supplementary Figure S3. ZNF498 interacts with p53, represses p53 transcriptional activity and inhibits p53 Ser46 phosphorylation under DNA damage conditions. Supplementary Figure S4. ZNF498 has no effect on p53 Ser46 phosphorylation in HCC cells with knockdown of DYRK2, ATM, HIPK1 and p38. Supplementary Figure S5. ZNF498 does not interact with p53INP1. Supplementary Figure S6. ZNF498 promotes HCC cell growth in vitro. Supplementary Figure S7. p53 expression was identified in HepG2 cells with stable knockout of p53. Supplementary Figure S8. The correlation between ZNF498 overexpression and different p53 statuses in HCC tissues. Supplementary Figure S9. ZNF498 represses p53-mediated apoptosis. Supplementary Figure S10. ZNF498 represses ferroptosis. Supplementary Figure S11. ZNF498 represses p53 activity and apoptosis by inhibiting p53 Ser46 phosphorylation. [file 13046_2022_2288_MOESM1_ESM.zip › Supplementary Fig.S1.jpg]

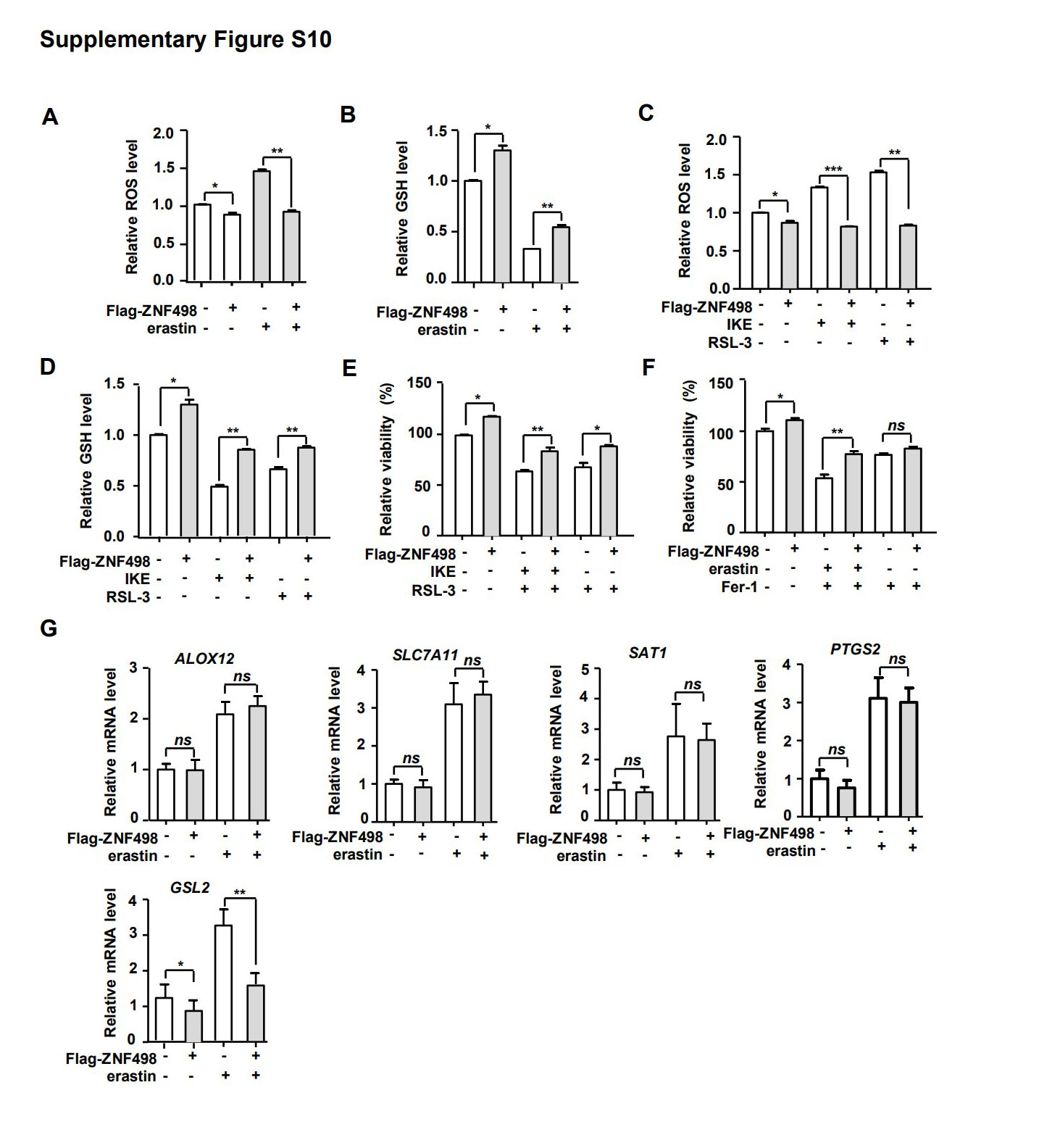

Supplement: Supplementary file 1 — Additional file 1: Supplementary Figure S1. Anti-ZNF498 antibody specifically recognizes ZNF498. Supplementary Figure S2. ZNF498 promotes the initiation of DEN-induced HCC. Supplementary Figure S3. ZNF498 interacts with p53, represses p53 transcriptional activity and inhibits p53 Ser46 phosphorylation under DNA damage conditions. Supplementary Figure S4. ZNF498 has no effect on p53 Ser46 phosphorylation in HCC cells with knockdown of DYRK2, ATM, HIPK1 and p38. Supplementary Figure S5. ZNF498 does not interact with p53INP1. Supplementary Figure S6. ZNF498 promotes HCC cell growth in vitro. Supplementary Figure S7. p53 expression was identified in HepG2 cells with stable knockout of p53. Supplementary Figure S8. The correlation between ZNF498 overexpression and different p53 statuses in HCC tissues. Supplementary Figure S9. ZNF498 represses p53-mediated apoptosis. Supplementary Figure S10. ZNF498 represses ferroptosis. Supplementary Figure S11. ZNF498 represses p53 activity and apoptosis by inhibiting p53 Ser46 phosphorylation. [file 13046_2022_2288_MOESM1_ESM.zip › Supplementary Fig.S10.jpg]

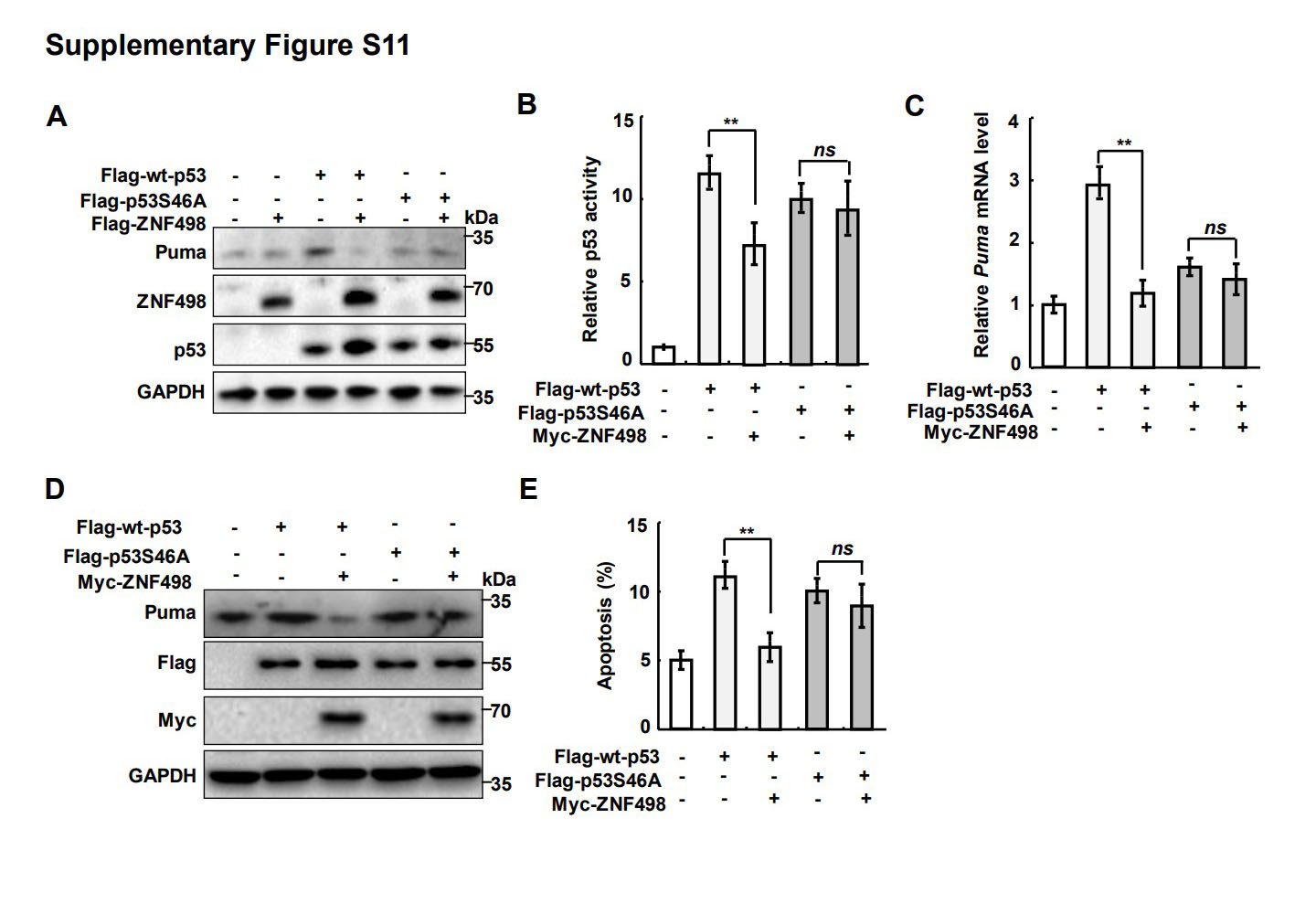

Supplement: Supplementary file 1 — Additional file 1: Supplementary Figure S1. Anti-ZNF498 antibody specifically recognizes ZNF498. Supplementary Figure S2. ZNF498 promotes the initiation of DEN-induced HCC. Supplementary Figure S3. ZNF498 interacts with p53, represses p53 transcriptional activity and inhibits p53 Ser46 phosphorylation under DNA damage conditions. Supplementary Figure S4. ZNF498 has no effect on p53 Ser46 phosphorylation in HCC cells with knockdown of DYRK2, ATM, HIPK1 and p38. Supplementary Figure S5. ZNF498 does not interact with p53INP1. Supplementary Figure S6. ZNF498 promotes HCC cell growth in vitro. Supplementary Figure S7. p53 expression was identified in HepG2 cells with stable knockout of p53. Supplementary Figure S8. The correlation between ZNF498 overexpression and different p53 statuses in HCC tissues. Supplementary Figure S9. ZNF498 represses p53-mediated apoptosis. Supplementary Figure S10. ZNF498 represses ferroptosis. Supplementary Figure S11. ZNF498 represses p53 activity and apoptosis by inhibiting p53 Ser46 phosphorylation. [file 13046_2022_2288_MOESM1_ESM.zip › Supplementary Fig.S11.jpg]

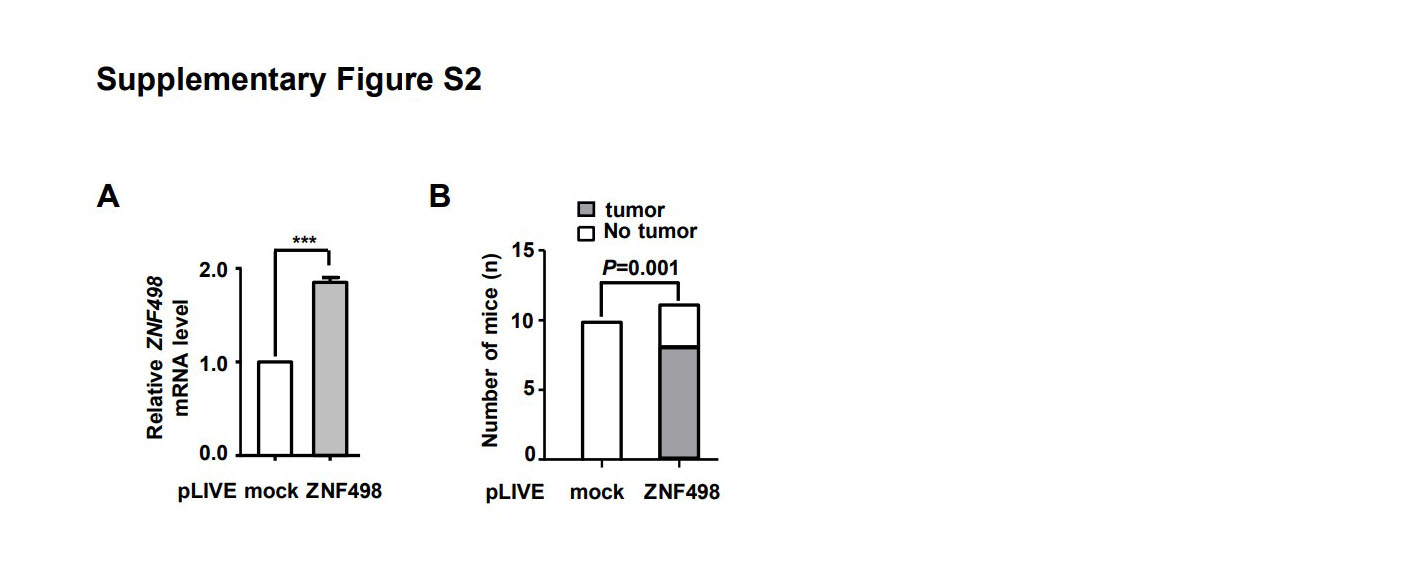

Supplement: Supplementary file 1 — Additional file 1: Supplementary Figure S1. Anti-ZNF498 antibody specifically recognizes ZNF498. Supplementary Figure S2. ZNF498 promotes the initiation of DEN-induced HCC. Supplementary Figure S3. ZNF498 interacts with p53, represses p53 transcriptional activity and inhibits p53 Ser46 phosphorylation under DNA damage conditions. Supplementary Figure S4. ZNF498 has no effect on p53 Ser46 phosphorylation in HCC cells with knockdown of DYRK2, ATM, HIPK1 and p38. Supplementary Figure S5. ZNF498 does not interact with p53INP1. Supplementary Figure S6. ZNF498 promotes HCC cell growth in vitro. Supplementary Figure S7. p53 expression was identified in HepG2 cells with stable knockout of p53. Supplementary Figure S8. The correlation between ZNF498 overexpression and different p53 statuses in HCC tissues. Supplementary Figure S9. ZNF498 represses p53-mediated apoptosis. Supplementary Figure S10. ZNF498 represses ferroptosis. Supplementary Figure S11. ZNF498 represses p53 activity and apoptosis by inhibiting p53 Ser46 phosphorylation. [file 13046_2022_2288_MOESM1_ESM.zip › Supplementary Fig.S2.jpg]

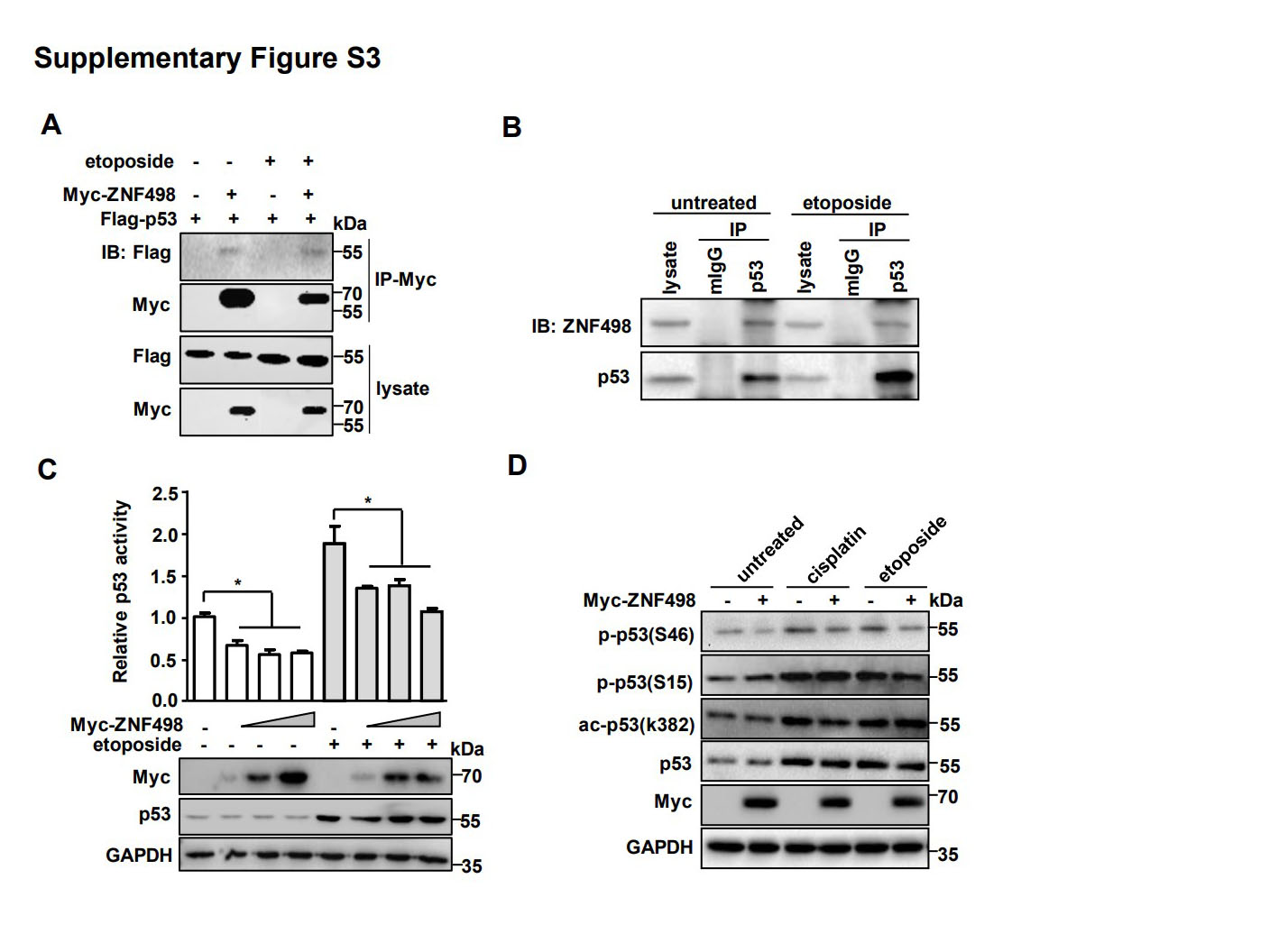

Supplement: Supplementary file 1 — Additional file 1: Supplementary Figure S1. Anti-ZNF498 antibody specifically recognizes ZNF498. Supplementary Figure S2. ZNF498 promotes the initiation of DEN-induced HCC. Supplementary Figure S3. ZNF498 interacts with p53, represses p53 transcriptional activity and inhibits p53 Ser46 phosphorylation under DNA damage conditions. Supplementary Figure S4. ZNF498 has no effect on p53 Ser46 phosphorylation in HCC cells with knockdown of DYRK2, ATM, HIPK1 and p38. Supplementary Figure S5. ZNF498 does not interact with p53INP1. Supplementary Figure S6. ZNF498 promotes HCC cell growth in vitro. Supplementary Figure S7. p53 expression was identified in HepG2 cells with stable knockout of p53. Supplementary Figure S8. The correlation between ZNF498 overexpression and different p53 statuses in HCC tissues. Supplementary Figure S9. ZNF498 represses p53-mediated apoptosis. Supplementary Figure S10. ZNF498 represses ferroptosis. Supplementary Figure S11. ZNF498 represses p53 activity and apoptosis by inhibiting p53 Ser46 phosphorylation. [file 13046_2022_2288_MOESM1_ESM.zip › Supplementary Fig.S3.jpg]

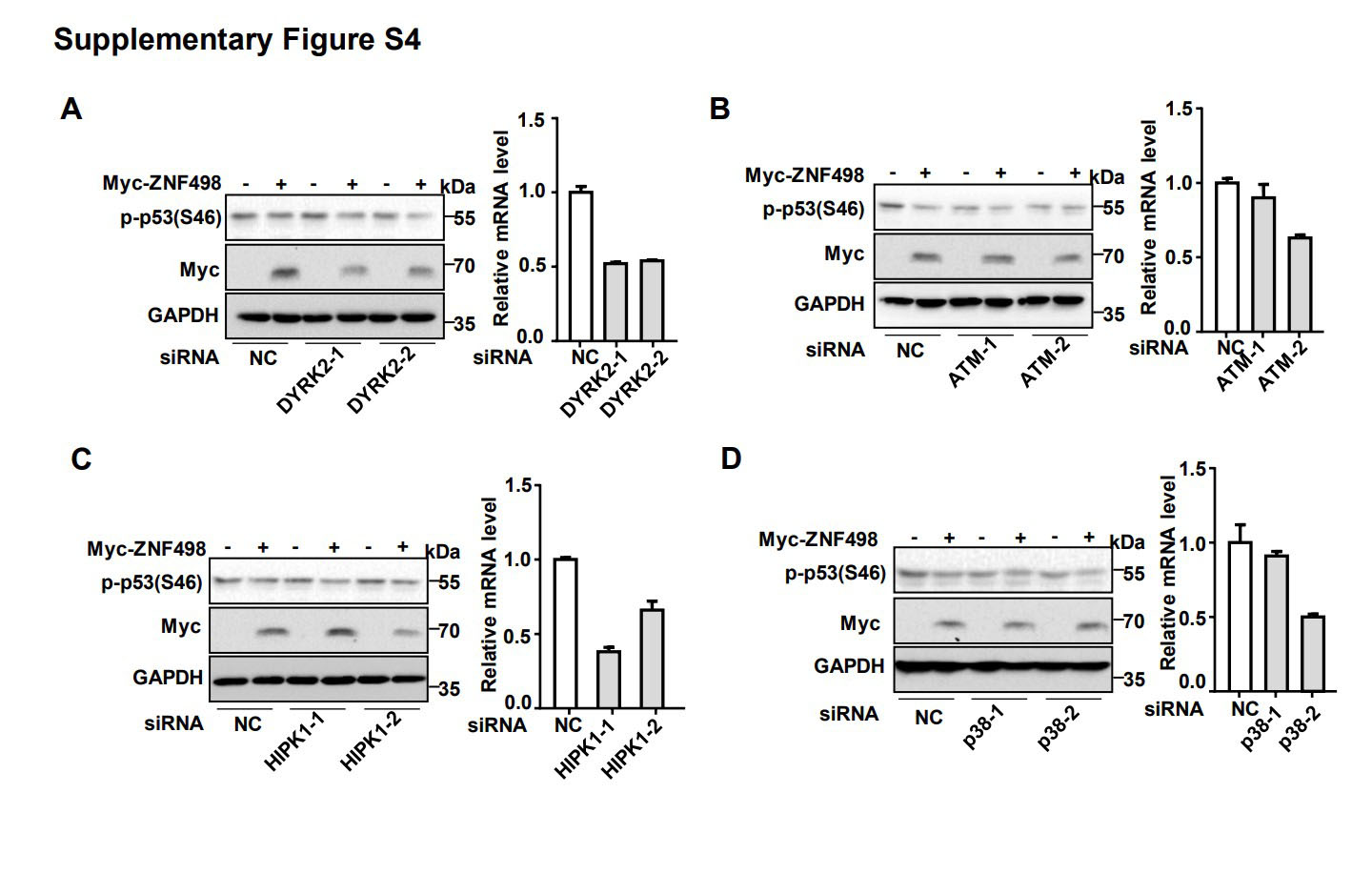

Supplement: Supplementary file 1 — Additional file 1: Supplementary Figure S1. Anti-ZNF498 antibody specifically recognizes ZNF498. Supplementary Figure S2. ZNF498 promotes the initiation of DEN-induced HCC. Supplementary Figure S3. ZNF498 interacts with p53, represses p53 transcriptional activity and inhibits p53 Ser46 phosphorylation under DNA damage conditions. Supplementary Figure S4. ZNF498 has no effect on p53 Ser46 phosphorylation in HCC cells with knockdown of DYRK2, ATM, HIPK1 and p38. Supplementary Figure S5. ZNF498 does not interact with p53INP1. Supplementary Figure S6. ZNF498 promotes HCC cell growth in vitro. Supplementary Figure S7. p53 expression was identified in HepG2 cells with stable knockout of p53. Supplementary Figure S8. The correlation between ZNF498 overexpression and different p53 statuses in HCC tissues. Supplementary Figure S9. ZNF498 represses p53-mediated apoptosis. Supplementary Figure S10. ZNF498 represses ferroptosis. Supplementary Figure S11. ZNF498 represses p53 activity and apoptosis by inhibiting p53 Ser46 phosphorylation. [file 13046_2022_2288_MOESM1_ESM.zip › Supplementary Fig.S4.jpg]

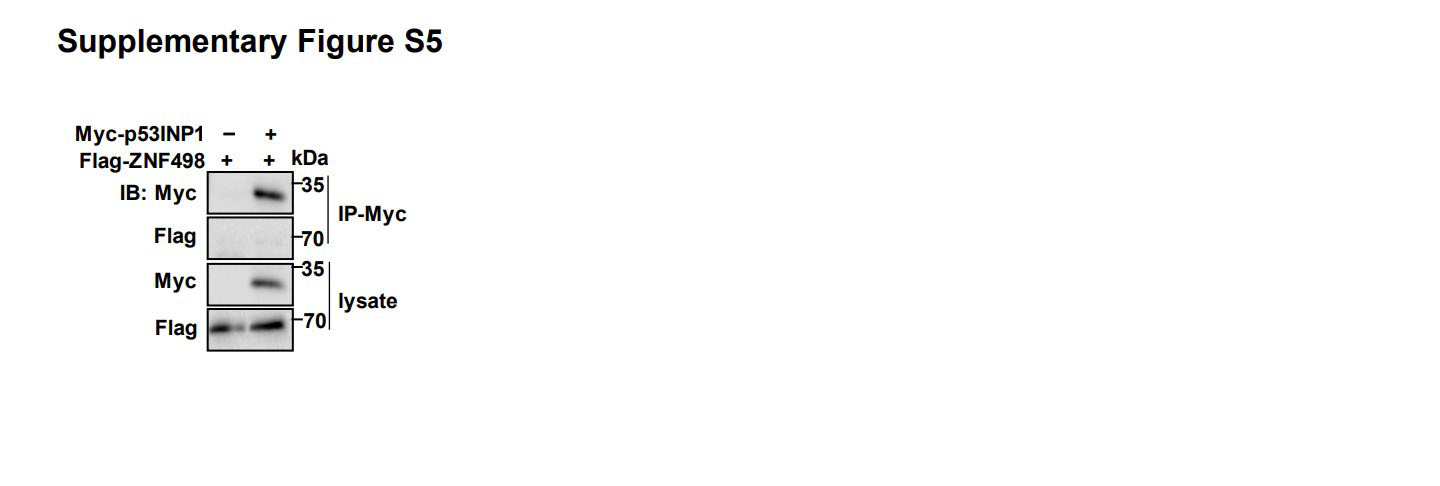

Supplement: Supplementary file 1 — Additional file 1: Supplementary Figure S1. Anti-ZNF498 antibody specifically recognizes ZNF498. Supplementary Figure S2. ZNF498 promotes the initiation of DEN-induced HCC. Supplementary Figure S3. ZNF498 interacts with p53, represses p53 transcriptional activity and inhibits p53 Ser46 phosphorylation under DNA damage conditions. Supplementary Figure S4. ZNF498 has no effect on p53 Ser46 phosphorylation in HCC cells with knockdown of DYRK2, ATM, HIPK1 and p38. Supplementary Figure S5. ZNF498 does not interact with p53INP1. Supplementary Figure S6. ZNF498 promotes HCC cell growth in vitro. Supplementary Figure S7. p53 expression was identified in HepG2 cells with stable knockout of p53. Supplementary Figure S8. The correlation between ZNF498 overexpression and different p53 statuses in HCC tissues. Supplementary Figure S9. ZNF498 represses p53-mediated apoptosis. Supplementary Figure S10. ZNF498 represses ferroptosis. Supplementary Figure S11. ZNF498 represses p53 activity and apoptosis by inhibiting p53 Ser46 phosphorylation. [file 13046_2022_2288_MOESM1_ESM.zip › Supplementary Fig.S5.jpg]

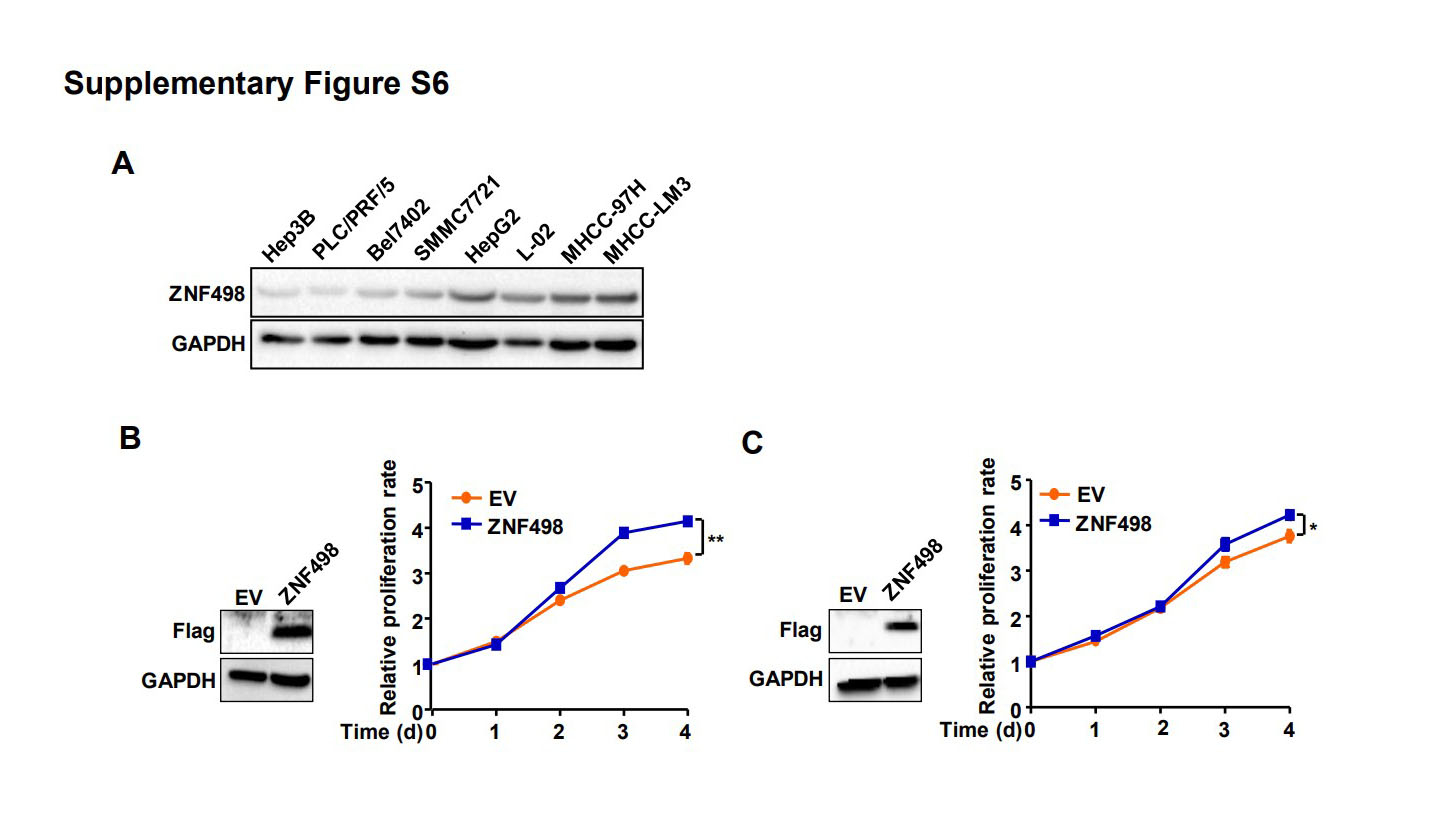

Supplement: Supplementary file 1 — Additional file 1: Supplementary Figure S1. Anti-ZNF498 antibody specifically recognizes ZNF498. Supplementary Figure S2. ZNF498 promotes the initiation of DEN-induced HCC. Supplementary Figure S3. ZNF498 interacts with p53, represses p53 transcriptional activity and inhibits p53 Ser46 phosphorylation under DNA damage conditions. Supplementary Figure S4. ZNF498 has no effect on p53 Ser46 phosphorylation in HCC cells with knockdown of DYRK2, ATM, HIPK1 and p38. Supplementary Figure S5. ZNF498 does not interact with p53INP1. Supplementary Figure S6. ZNF498 promotes HCC cell growth in vitro. Supplementary Figure S7. p53 expression was identified in HepG2 cells with stable knockout of p53. Supplementary Figure S8. The correlation between ZNF498 overexpression and different p53 statuses in HCC tissues. Supplementary Figure S9. ZNF498 represses p53-mediated apoptosis. Supplementary Figure S10. ZNF498 represses ferroptosis. Supplementary Figure S11. ZNF498 represses p53 activity and apoptosis by inhibiting p53 Ser46 phosphorylation. [file 13046_2022_2288_MOESM1_ESM.zip › Supplementary Fig.S6.jpg]

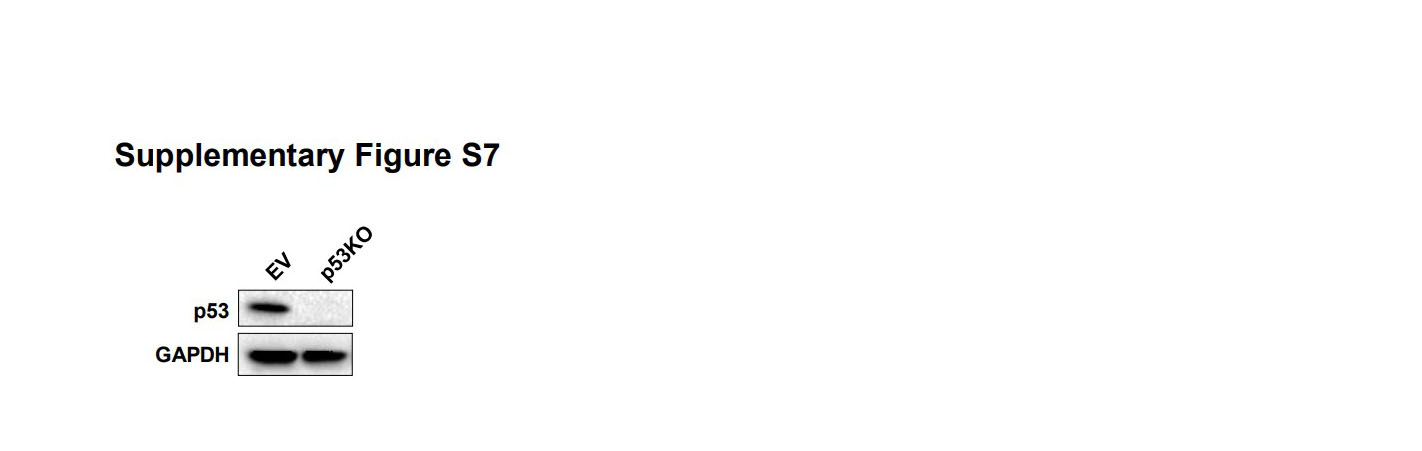

Supplement: Supplementary file 1 — Additional file 1: Supplementary Figure S1. Anti-ZNF498 antibody specifically recognizes ZNF498. Supplementary Figure S2. ZNF498 promotes the initiation of DEN-induced HCC. Supplementary Figure S3. ZNF498 interacts with p53, represses p53 transcriptional activity and inhibits p53 Ser46 phosphorylation under DNA damage conditions. Supplementary Figure S4. ZNF498 has no effect on p53 Ser46 phosphorylation in HCC cells with knockdown of DYRK2, ATM, HIPK1 and p38. Supplementary Figure S5. ZNF498 does not interact with p53INP1. Supplementary Figure S6. ZNF498 promotes HCC cell growth in vitro. Supplementary Figure S7. p53 expression was identified in HepG2 cells with stable knockout of p53. Supplementary Figure S8. The correlation between ZNF498 overexpression and different p53 statuses in HCC tissues. Supplementary Figure S9. ZNF498 represses p53-mediated apoptosis. Supplementary Figure S10. ZNF498 represses ferroptosis. Supplementary Figure S11. ZNF498 represses p53 activity and apoptosis by inhibiting p53 Ser46 phosphorylation. [file 13046_2022_2288_MOESM1_ESM.zip › Supplementary Fig.S7.jpg]

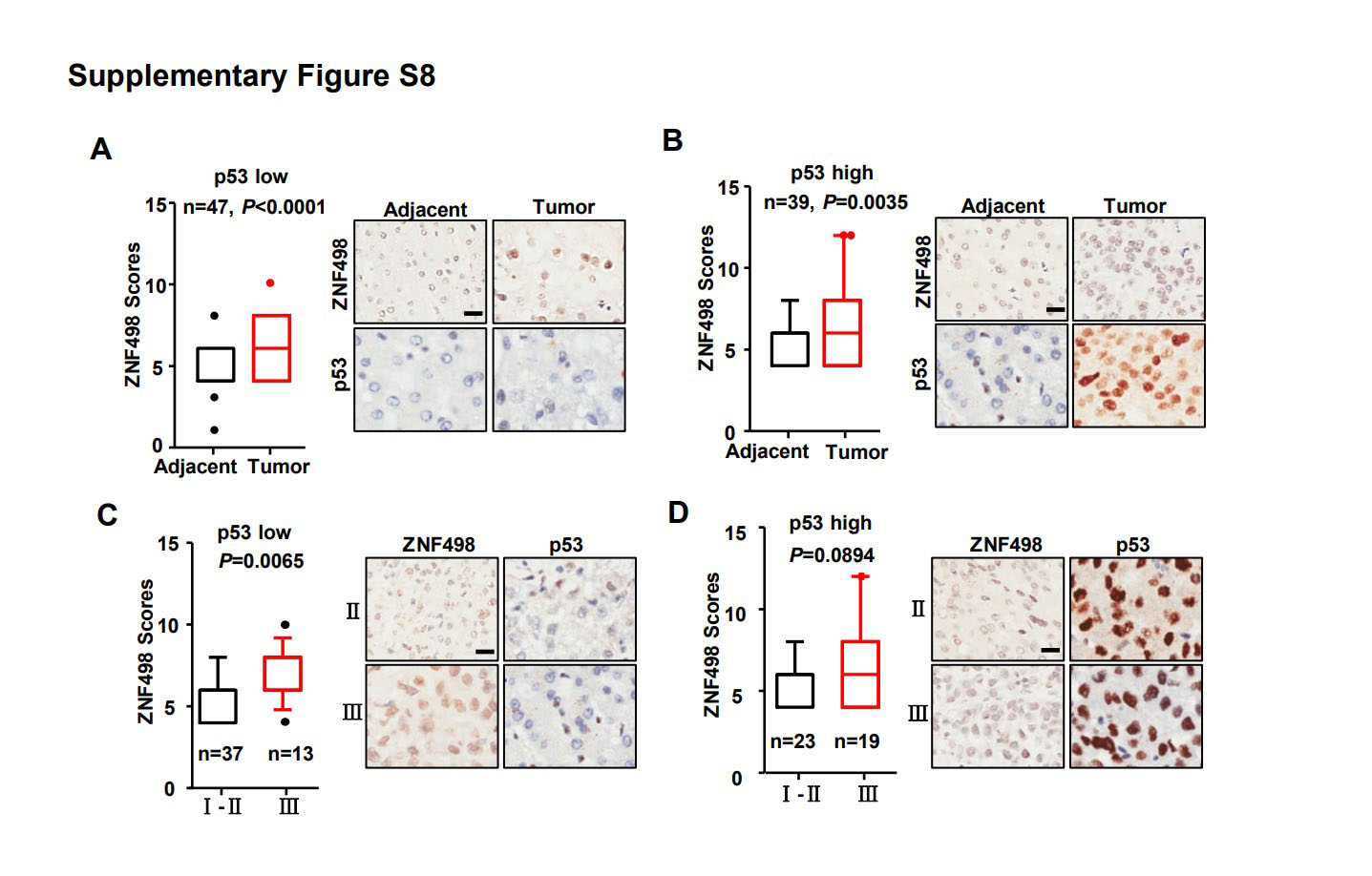

Supplement: Supplementary file 1 — Additional file 1: Supplementary Figure S1. Anti-ZNF498 antibody specifically recognizes ZNF498. Supplementary Figure S2. ZNF498 promotes the initiation of DEN-induced HCC. Supplementary Figure S3. ZNF498 interacts with p53, represses p53 transcriptional activity and inhibits p53 Ser46 phosphorylation under DNA damage conditions. Supplementary Figure S4. ZNF498 has no effect on p53 Ser46 phosphorylation in HCC cells with knockdown of DYRK2, ATM, HIPK1 and p38. Supplementary Figure S5. ZNF498 does not interact with p53INP1. Supplementary Figure S6. ZNF498 promotes HCC cell growth in vitro. Supplementary Figure S7. p53 expression was identified in HepG2 cells with stable knockout of p53. Supplementary Figure S8. The correlation between ZNF498 overexpression and different p53 statuses in HCC tissues. Supplementary Figure S9. ZNF498 represses p53-mediated apoptosis. Supplementary Figure S10. ZNF498 represses ferroptosis. Supplementary Figure S11. ZNF498 represses p53 activity and apoptosis by inhibiting p53 Ser46 phosphorylation. [file 13046_2022_2288_MOESM1_ESM.zip › Supplementary Fig.S8.jpg]

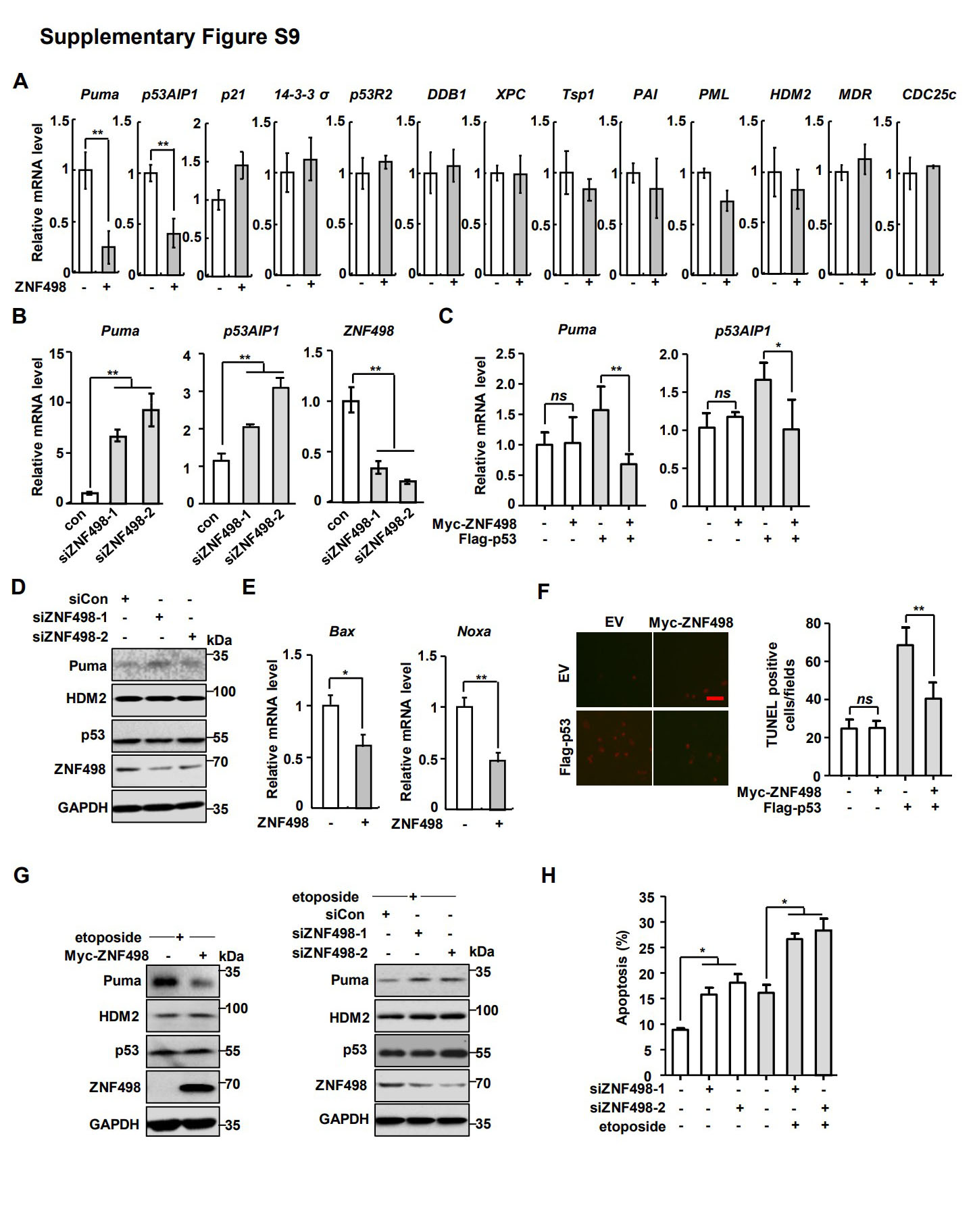

Supplement: Supplementary file 1 — Additional file 1: Supplementary Figure S1. Anti-ZNF498 antibody specifically recognizes ZNF498. Supplementary Figure S2. ZNF498 promotes the initiation of DEN-induced HCC. Supplementary Figure S3. ZNF498 interacts with p53, represses p53 transcriptional activity and inhibits p53 Ser46 phosphorylation under DNA damage conditions. Supplementary Figure S4. ZNF498 has no effect on p53 Ser46 phosphorylation in HCC cells with knockdown of DYRK2, ATM, HIPK1 and p38. Supplementary Figure S5. ZNF498 does not interact with p53INP1. Supplementary Figure S6. ZNF498 promotes HCC cell growth in vitro. Supplementary Figure S7. p53 expression was identified in HepG2 cells with stable knockout of p53. Supplementary Figure S8. The correlation between ZNF498 overexpression and different p53 statuses in HCC tissues. Supplementary Figure S9. ZNF498 represses p53-mediated apoptosis. Supplementary Figure S10. ZNF498 represses ferroptosis. Supplementary Figure S11. ZNF498 represses p53 activity and apoptosis by inhibiting p53 Ser46 phosphorylation. [file 13046_2022_2288_MOESM1_ESM.zip › Supplementary Fig.S9.jpg]
