# Supplementary material for: ZNF498 promotes hepatocellular carcinogenesis by suppressing p53-mediated apoptosis and ferroptosis via the attenuation of p53 Ser46 phosphorylation
Source: J Exp Clin Cancer Res. 2022 Feb 28;41:79. doi: 10.1186/s13046-022-02288-3 (PMC8883630; doi:10.1186/s13046-022-02288-3)
Supplement: Supplementary file 2 — Additional file 2: Table S1. Antibodies information. [file 13046_2022_2288_MOESM2_ESM.docx]

**Table S1.** Antibodies information

| Antibody | Catalog | Company |
| --- | --- | --- |
| p53 | OP43L | Calbiochem |
| phosphorylated p53 (Ser 15) | 9284S | Cell Signaling Technology |
| acetylated p53 (K382/383) | 2525S | Cell Signaling Technology |
| phosphorylated p53 (Ser 46) | GR116931-5 | Abcam |
| puma | 4976S | Cell Signaling Technology |
| p53R2 | A-5 | Santa Cruz |
| HDM2 | EX15828 | Huaxingbio, Beijing, China |
| p53DINP1 | ab202026 | Abcam |
| GAPDH | 60004-1 | Proteintac |
| Myc-HRP | M047-3 | Medical Biological Laboratories |
| Flag-HRP | SL12445 | Sigma-Aldrich |
