# Supplementary material for: ZNF498 promotes hepatocellular carcinogenesis by suppressing p53-mediated apoptosis and ferroptosis via the attenuation of p53 Ser46 phosphorylation
Source: J Exp Clin Cancer Res. 2022 Feb 28;41:79. doi: 10.1186/s13046-022-02288-3 (PMC8883630; doi:10.1186/s13046-022-02288-3)
Supplement: Supplementary file 3 — Additional file 3: Table S2. siRNA sequences. [file 13046_2022_2288_MOESM3_ESM.docx]

**Table S2.** siRNA sequences

| siRNA | sequences（5'-3'） |
| --- | --- |
| p38 MAPK-1 | GAAGCTCTCCAGACCATTT |
| p38 MAPK-2 | TATCCATTCAGCTAACGTTCT |
| PKCδ-1 | CCGUUCCUGCGCAUCUCCUUCAAUU |
| PKCδ-2 | CCACGAGUUUAUCGCCACCTT |
| DYRK2-1 | GGUGCUAUCACAUCUAUAU |
| DYRK2-2 | CCACGATCACGTGGCTTACAGGTAT |
| ATM-1 | AAGGCTATTCAGTGTGCGAGA |
| ATM-2 | UGGUGCUAUUUACGGAGCU |
| HIPK2-1 | CCAGGTGAACATGACGACAGA |
| HIPK2-2  p53DINP1-1  p53DINP1-2 | AAGCGTCGGGTGAATATGTAT  GCGCCATGTTTCTCAAAGTTT  GCCTTCATAATCAAACAGCTT |
| ZNF498-1 | AGCGCACCAUCACAUCUAATT |
| ZNF498-2 | CCCACGAAGAGAAGUCUUATT |
