# Supplementary material for: ZNF498 promotes hepatocellular carcinogenesis by suppressing p53-mediated apoptosis and ferroptosis via the attenuation of p53 Ser46 phosphorylation
Source: J Exp Clin Cancer Res. 2022 Feb 28;41:79. doi: 10.1186/s13046-022-02288-3 (PMC8883630; doi:10.1186/s13046-022-02288-3)
Supplement: Supplementary file 5 — Additional file 5: Table S4. Primer sequences. [file 13046_2022_2288_MOESM5_ESM.docx]

**Table S4.** Primer sequences

| Gene | Forward（5'-3'） | Reverse（5'-3'） | |  |
| --- | --- | --- | --- | --- |
| ZNF498 mus | CTGCCCCGAGAGTTCTACAC | GGATGCCTGATTGCCACGAT- | |  |
| β-actin mus | GTGACGTTGACATCCGTAAAGA | GCCGGACTCATCGTACTCC | |  |
| ZNF498 | GCAGCAGTTGGGTATTCCTGT | | GCCGAAAAGTCTCTGGACTAGG | |
| Puma | GACCTCAACGCACAGTA | | CTAATTGGGCTCCATCT | |
| p53AIP1 | TCTTCCTCTGAGGCGAGCT | | AGGTGTGTGTGTCTGAGCCC | |
| p21 | CAGCATGACAGATTTCTACC | | GAGACTAAGGCAGAAGATGT | |
| 14-3-3σ | GGCCATGGACATCAGCAAGA | | CGATGGTCTTGGCCGAG | |
| Gadd45 | GGATGCCCTGGAGGAAGTG | | GGTCGACGTTGAGCAGCTTG | |
| p53R2 | TGGCTTCGTCGTTGCGAGCG | | TCTGAAGATGATCTCCCGGCCT | |
| DDB1 | CTGAACCCATGCTGTGATTG | | AAACTCGGATCTCGCTCTTC | |
| XPC | ACGGGCCCAAGAGTGAGGC | | TTGAGGCCAGGAGGCAGCCA | |
| TSP1 | CCCGTGGTCATCTTGTTCTGT | | TTTCTTGCAGGCTTTGGTCTCC | |
| PAI | CTCCTGGTTCTGCCCAAGTT | | GAGAGGCTTTGGTCTGAAAG | |
| PML | CGCCCTGGATAACGTCTTTTT | | TCCACAATCTGCCGGTACAC | |
| HDM2 | ATCTTGGCCAGTATATTATG | | GTTCCTGTAGATCATGGTAT | |
| MDR | ATAATGCGACAGGAGATAGG | | CCAAAATCACAAGGGTTAGC | |
| CDC25c | GAACAGGCCAAGACTGAAGC | | GCCCCTGGTTAGAATCTTCC | |
| GAPDH | GGGAAGGTGAAGGTCGGAGT | | TTGAGGTCAATGAAGGGGTCA | |
| ATM | ATCTGCTGCCGTCAACTAGAA | | GATCTCGAATCAGGCGCTTAAA | |
| MAPK | TACACCAACCTCTCGTACATCG | | CATGTCTGAAGCGCAGTAAGATT | |
| HIPK2 | CCCGTGTACGAAGGTATGGC | | AGTTGGAACTCGGCTCTATTTTC | |
| DYRK2 | CAGTGCTCACGACACAACCA | | CCGTCTATGAATGCTGTCCAG | |
| PKCδ | GTGCAGAAGAAGCCGACCAT | | CCCGCATTAGCACAATCTGGA | |
| p53DINP1 | TTCCTCCAACCAAGAACCAGA | | GCTCAGTAGGTGACTCTTCACT | |
| SLC7A11 | TCATTGGAGCAGGAATCTTCA | | TTCAGCATAAGACAAAGCTCCA | |
| ALOX12 | ACCCGTGGATTGGCAAGTTAT | | TGCAACCTGGCTTAGATTCTTC | |
| PTGS2 | CTTCACGCATCAGTTTTTCAAG | | TCACCGTAAATATGATTTAAGTCCAC | |
| SAT1 | CAGTGTCACCATCTGAGCCC | | GTAGGGGGTTTAACTGGGGG | |
| Bax | GGCTGGACATTGGACTTC | | CTTCCAGATGGTGAGTGAGG | |
| Noxa | ACCAAGCCGGATTTGCGATT | | ACTTGCACTTGTTCCTCGTGG | |
