# Supplementary material for: ZNF498 promotes hepatocellular carcinogenesis by suppressing p53-mediated apoptosis and ferroptosis via the attenuation of p53 Ser46 phosphorylation
Source: J Exp Clin Cancer Res. 2022 Feb 28;41:79. doi: 10.1186/s13046-022-02288-3 (PMC8883630; doi:10.1186/s13046-022-02288-3)
Supplement: Supplementary file 6 — Additional file 6: Table S5. Correlation between expression levels of ZNF498 and clinicopathological characteristics in patients with HCC. [file 13046_2022_2288_MOESM6_ESM.docx]

**Table S5.** Correlation between expression levels of ZNF498 and clinicopathological characteristics in patients with HCC.

| Characteristics | ZNF498 expression | | *P* |
| --- | --- | --- | --- |
|  | High low | |  |
| Age |  |  | 0.349 |
| <50 | 8 | 27 |  |
| ≥50 | 19 | 38 |  |
| Sex |  |  | 0.472 |
| Male | 23 | 59 |  |
| Female | 4 | 6 |  |
| Liver cirrhosis |  |  | 0.815 |
| Absent | 16 | 41 |  |
| Present | 11 | 24 |  |
| Tumor multiplicity |  |  | 0.215 |
| Single | 25 | 52 |  |
| Multiple | 2 | 13 |  |
| Maximal Tumor size (cm) |  |  | 1.000 |
| <5 | 11 | 28 |  |
| ≥5 | 16 | 37 |  |
| Histological grade |  |  | **0.003** |
| I | 1 | 2 |  |
| II | 10 | 47 |  |
| III | 16 | 16 |  |
| TNM stage |  |  | 0.34 |
| I | 6 | 6 |  |
| II | 8 | 25 |  |
| III | 11 | 31 |  |
| IV | 2 | 3 |  |
| Portal vein thrombosis |  |  | 0.669 |
| Absence | 26 | 59 |  |
| Gross | 1 | 6 |  |

ZNF498 low (n = 65), ZNF498 high (n = 27)
